# Immuno-Mechanical Signaling Network Integration in Temporomandibular Joint Pathology: A TMID Conceptual Framework

**DOI:** 10.3390/ijms27083363

**Published:** 2026-04-09

**Authors:** Hyoung-Jun Kim, Jae-Hong Kim, Jong-Il Yun

**Affiliations:** 1 OFP Dental Clinic, Seoul 06237, Republic of Korea; 2 Department of Oral Medicine and Oral Diagnosis, School of Dentistry, Seoul National University, Seoul 03080, Republic of Korea; oralyun@naver.com; 3 Seoultop Dental Clinic, Seoul 12218, Republic of Korea; flavan@naver.com; 4 Yeon Dental Clinic, Seoul 04363, Republic of Korea

**Keywords:** temporomandibular disorders, temporomandibular immunologic disease, macrophage polarization, Th17 cells, regulatory T cells, mechanotransduction, subchondral bone remodeling

## Abstract

Temporomandibular disorders (TMDs) are multifactorial conditions traditionally attributed to excessive mechanical loading on the temporomandibular joint, leading to clinical manifestations ranging from joint sounds to structural deformation. Contributing factors include trauma, occlusal abnormalities, psychological stress, and bruxism. However, immune and molecular alterations associated with early disease activity are not systematically integrated into structure-centered TMD frameworks. Emerging evidence indicates that temporomandibular joint osteoarthritis (TMJOA) involves activation of innate immunity caused by damage-associated molecular patterns (DAMPs) generated through mechanical loading, together with non-antigen-specific adaptive immune responses, including macrophage polarization and T helper 17 (Th17) and regulatory T (Treg) cell imbalance. Inflammatory and mechanical inputs converge through shared signaling modules and mechanoresponsive transcriptional programs, promoting extracellular matrix degradation, fibrotic remodeling, and subchondral bone remodeling. This review synthesizes the current immunopathological and mechanobiological evidence and introduces temporomandibular immunologic disease (TMID) as a mechanism-oriented framework, characterized by a reinforcing cycle between mechanically induced tissue damage and immune activation within the temporomandibular joint (TMJ) microenvironment. TMID complements TMJOA and Diagnostic Criteria for Temporomandibular Disorders (DC/TMD) structural diagnostic categories while excluding antigen-specific autoimmune arthritides such as rheumatoid arthritis, thus functioning as a mechanistic overlay framework for the integration of immuno-mechanical signaling networks in immune-active, mechanically driven TMJ pathology.

## 1. Introduction

### Background and Conceptual Framework of Temporomandibular Immunologic Disease

The immune system detects not only infectious threats but also danger signals arising from cellular injury and disruption of tissue homeostasis, responding to both damage-associated molecular patterns (DAMPs) and pathogen-associated molecular patterns (PAMPs) [1,2]. Repetitive or persistent exposure to these stimuli can sequentially and reciprocally activate innate and adaptive immune responses, thereby amplifying inflammatory reactions [1,2,3,4]. This danger-sensing paradigm provides a conceptual framework for understanding how sterile tissue injury can initiate and sustain inflammation in the absence of overt infection.

In the temporomandibular joint (TMJ), repetitive mechanical loading induces the release of DAMPs from the synovium, articular disc, and cartilage, including extracellular matrix (ECM) fragments and modified or denatured proteins [3,5]. These DAMPs are recognized by pattern recognition receptors (PRRs), including Toll-like receptors (TLRs), and can establish an inflammatory microenvironment under non-infectious conditions [4]. During this process, synovial macrophages adopt a classically activatedM1 (pro-inflammatory) phenotype characterized by increased cytokine production. Concurrently, chemokine axes derived from activated synovial cells promote the recruitment of inflammatory cells and T lymphocytes [6,7]. These observations indicate that mechanical tissue injury in the TMJ is coupled to immune activation, in part through DAMP–PRR signaling.

At the cellular level, nuclear factor-κB (NF-κB) signaling is consistently activated, while mitogen-activated protein kinase (MAPK) pathways are engaged in a cell type-dependent manner. These cascades increase the expression of proinflammatory cytokines, matrix metalloproteinases (MMPs), and a disintegrin and metalloproteinase with thrombospondin motifs (ADAMTS) family proteases, thereby promoting synovitis and extracellular matrix degradation [8,9]. In parallel, the Janus kinase–signal transducer and activator of transcription (JAK–STAT) axis amplifies cytokine-driven signaling in synovial immune cells and contributes to both synovial inflammation and cartilage destruction [9,10]. Excessive mechanical loading further modulates integrin–focal adhesion kinase (FAK) signaling and alters extracellular matrix remodeling programs in TMJ fibrocartilage. In addition, Yes-associated protein (YAP)-mediated mechanosensing—together with the transforming growth factor-β (TGF-β)–Ras homolog family member A (RhoA)/Rho-associated coiled-coil-containing protein kinase (ROCK)–actin–myocardin-related transcription factor (MRTF) axis—has been implicated in myofibroblast programming and fibrotic responses [11,12]. Mechanical overload may also promote macrophage and osteoclast activation, contributing to imbalances in subchondral bone resorption and remodeling [13]. Collectively, these findings indicate that inflammatory and mechanical inputs converge on partially shared signaling modules and transcriptional programs.

On this basis, we propose temporomandibular immunologic disease (TMID) as a pathophysiology-oriented conceptual framework. TMID reinterprets the temporomandibular disorder (TMD) spectrum, including temporomandibular joint osteoarthritis (TMJOA), as a continuum integrating immune-mediated mechanisms with established structural and degenerative paradigms. Importantly, TMID complements rather than replaces existing diagnostic classifications. In this framework, TMID is proposed as a disease-activity-oriented, immuno-mechanical interpretive framework that organizes convergent immunologic and mechanotransductive pathways into a stage-linked progression model in immune-active, mechanically driven TMJ pathology, rather than a descriptive aggregation of molecular findings. Within this continuum, DAMP-mediated innate immune activation induced by mechanical overload interacts with non-antigen-specific adaptive immune responses. These processes converge on tissue remodeling pathways and amplify damage in a feed-forward manner. This amplification is coordinated through crosstalk among inflammatory signaling axes, including NF-κB and JAK–STAT; the MAPK pathway, which responds to both inflammatory and mechanical inputs; and mechanotransductive pathways such as integrin–FAK–extracellular signal-regulated kinase (ERK)/phosphoinositide 3-kinase (PI3K) and YAP/ROCK–MRTF signaling [5,8,10,11,12]. For conceptual clarity, the provisional working stages of TMID are presented in Table 1.

TMID is not proposed as a definitive diagnostic classification system with fixed thresholds. Rather, the stages presented here should be interpreted as working, hypothesis-generating constructs designed to support mechanistic interpretation and future empirical validation.

Despite expanding knowledge of individual pathways, a critical gap remains. There is no mechanistic, disease-activity-linked staging framework that systematically connects immune activation states with remodeling phenotypes and therapeutic timing in TMJ disease. Existing classifications primarily emphasize clinical and structural features, and may not adequately capture underlying pathogenic mechanisms or dynamic immune activity. TMID aims to bridge this gap by integrating immune–mechanical coupling with remodeling trajectories.

Accordingly, TMJOA may exhibit complex immunopathologic features alongside structural degeneration. Increased levels of interleukin-1β (IL-1β), tumor necrosis factor-α (TNF-α), and interleukin-6 (IL-6), together with a higher proportion of M1-like macrophages, have been reported during immune-active or progressive fibrotic–erosive stages [6,14]. In parallel, synovial chemokine axes promote T cell recruitment [7], accompanied by activation of the T helper 1 (Th1)/T helper 17 (Th17) axis and receptor activator of nuclear factor κB ligand (RANKL)-associated bone resorption [15,16]. Collectively, these findings support the interpretation of TMJOA as a degenerative phenotype characterized by immune activation and feed-forward amplification of tissue damage.

This positional assignment is intended as an interpretive framework rather than a fixed categorization. TMJOA likely encompasses heterogeneous immune activation states and may follow distinct trajectories depending on the magnitude, duration, and temporal dynamics of mechanical and inflammatory inputs. From this perspective, TMID emphasizes upstream mechanisms and immune–mechanical coupling as determinants of dynamic disease activity beyond structural endpoints.

In this review, “network integration” refers to the functional convergence and reciprocal amplification of inflammatory and mechanotransductive signaling pathways, rather than a mathematically formalized computational network model.

Importantly, within this framework, the presence of shared molecular pathways does not imply equivalence of disease frameworks, as disease identity is defined by the organization, interaction, and temporal dynamics of these pathways within specific anatomical and biomechanical contexts rather than shared cytokine profiles alone. While inflammatory and mechanotransductive signaling modules are broadly conserved across joint diseases, their integration and disease-level interpretation within the TMJ environment may differ substantially.

Immune activation in this framework is not assumed to be the primary initiating event in all cases, but is instead conceptualized as a context-dependent response that may become self-sustaining following repetitive mechanical injury. In this model, repetitive mechanical loading serves as an initial trigger, after which immune responses can become self-amplifying and contribute to disease progression within a reciprocal immuno-mechanical amplification loop. Therefore, the TMID framework establishes experimentally testable and falsifiable relationships linking immune activation, mechanical loading, and structural remodeling, thereby enabling quantitative and longitudinal validation. Importantly, while this framework does not yet define fixed diagnostic thresholds, it provides a foundation for future biomarker-based stratification and validation.

Importantly, although TMID shares multiple inflammatory mediators with autoimmune arthritides, overlap at the cytokine or effector pathway level does not imply equivalence of disease mechanisms, which are instead defined by initiating triggers, immune specificity, and systemic involvement rather than shared cytokine profiles alone. Rheumatoid arthritis is a systemic, antigen-specific autoimmune disease characterized by autoantibody production and multi-joint involvement, whereas TMID is conceptualized as a mechanically driven, locally amplified, non-antigen-specific immune-active condition of the TMJ microenvironment. Accordingly, TMID is not intended for application in patients with suspected or established autoimmune arthritides, and should be interpreted only within the context of mechanically driven TMJ pathology following appropriate clinical and serologic exclusion.

## 2. Rationale for TMID as an Immuno-Mechanical Network Model: Comparative and Anatomical Basis

### 2.1. Comparative Immunopathological and Mechanistic Features of Inflammatory and Degenerative Arthropathies

A comparative overview of initiating drivers, antigen specificity, immuno-mechanical coupling, dominant immune cell profiles, and therapeutic targets across rheumatoid arthritis (RA), psoriatic arthritis (PsA), OA, TMJOA, and TMID is summarized in Table 2.

Thus, shared cytokine networks do not define disease identity; rather, classification depends on initiating mechanisms, antigen specificity, and systemic versus local disease context.

Within this disease spectrum, RA represents a prototypical disorder driven by antigen-specific adaptive immunity, whereas PsA integrates innate and adaptive immune features driven by the interleukin-23–interleukin-17–tumor necrosis factor axis. In contrast, OA is primarily characterized by tissue damage driven by aging and mechanical loading, DAMP-mediated activation of innate immunity, and a low-grade inflammatory response.

TMJOA has been used primarily as a structural and imaging-based diagnostic term, but it may also be interpreted pathophysiologically as an immune-active phenotype characterized by non-antigen-specific adaptive immune activation, macrophage polarization, and T cell-related inflammatory signaling. However, current TMJOA classifications lack an explicit stage concept, which limits their ability to reflect disease continuity and therapeutic timing. In this context, TMID is thus proposed as a conceptual pathophysiological framework that enables an integrated, stage-specific interpretation in which innate immune activation induced by repetitive mechanical overload and non-antigen-specific adaptive immune responses are amplified through immuno-mechanical signaling axes.

### 2.2. Immunopathological Spectrum of Arthritic Diseases

#### 2.2.1. Immune-Dominant Arthritides: RA and PsA

RA is a prototypical immune-driven arthritic disease in which antigen-specific adaptive immune responses predominate, with CD4^+^ T cells and B cells infiltrating the synovium and mediating chronic synovitis and tissue destruction [17]. In contrast, TMID is not driven by antigen-specific adaptive immunity but is conceptualized as a mechanically initiated, locally amplified immune-active condition, in which innate immune activation and bystander adaptive responses are coupled to repetitive mechanical loading within the TMJ microenvironment. Although the clinical efficacy of immune-targeted therapies, including TNF-α, IL-6, and Janus kinase (JAK) inhibitors, has been established [18], conventional immune-centered models have limitations in explaining immune–mechanical interactions in an integrated manner.

PsA is understood as an inflammatory disease integrating innate and adaptive immune features, characterized by enthesis involvement and activation of the interleukin-23–interleukin-17–tumor necrosis factor axis, with reported involvement of Th17 cells, γδ T cells, and CD8^+^ T cells. Mechanical stress may amplify local inflammatory responses in PsA; however, the mechanisms through which repetitive mechanical loading contributes to chronic immune activation, and the relative contribution of immune–mechanical coupling, remain incompletely defined [19].

These immune-dominant patterns illustrate the utility of antigen-driven staging and underscore the need for a parallel, mechanism-linked staging concept in TMJ disease.

#### 2.2.2. Osteoarthritis as a Low-Grade, Innate-Dominant Arthropathy

Osteoarthritis (OA) is commonly understood as a disease in which aging, metabolic status, and abnormal mechanical loading lead to cumulative joint tissue damage, with ECM-derived DAMPs recognized by PRRs to induce innate immune responses and low-grade inflammation [20]. In parallel, the integrin–FAK signaling axis in chondrocytes functions as a principal mechanotransduction pathway conveying mechanical stimuli and ECM damage signals into intracellular responses [21].

In OA, the inflammatory response is generally low-grade and non-antigen-specific; although inflammatory signaling pathways such as NF-κB, MAPK, and JAK–STAT can be activated, their progression into a sustained immune-amplification circuitry appears to remain relatively limited [20,22,23]. Accordingly, OA is more often interpreted as a condition in which mechanical damage and low-grade inflammatory responses accumulate in parallel rather than being driven by adaptive immunity. Treatment likewise focuses on reducing mechanical loading and symptomatic management and remains limited in terms of an integrated pathophysiological framework that explains stage-specific disease progression [24].

This relatively limited immune-amplification pattern in OA may contrast with the immune-active TMJ phenotypes that informed the proposed TMID framework.

#### 2.2.3. TMJOA and TMID: Limitations of Structure-Based Diagnosis and Conceptual Expansion

In TMJOA, clinical phenotypes with more prominent immune activation and tissue damage have been reported in subsets of TMJOA and may extend beyond the low-grade inflammation typically described in OA. At this stage, chemokine-dependent T cell infiltration within a proinflammatory microenvironment; cytokine-mediated, non-antigen-specific adaptive immune activation (bystander activation); M1-like proinflammatory polarization of synovial macrophages; activation of the Th1/Th17 axis; and evidence of RANKL-associated bone resorption have been reported [6,7,15,25]. Furthermore, persistent tissue damage and inflammatory conditions may provide a conceptual basis for epitope spreading and potential expansion of immune targets over time [26]. However, as a structural and imaging-based diagnosis, TMJOA does not incorporate a staging framework that explains how immune activation states evolve and progress toward fibrotic remodeling.

TMID is proposed as a disease-activity-oriented, immuno-mechanical interpretive framework to contextualize these limitations by offering an integrative, stage-based interpretation of TMJ pathophysiology, in which DAMP-mediated innate immune activation induced by repetitive mechanical overload and non-antigen-specific adaptive immune responses may be mutually amplified through immuno-mechanical signaling axes. In this framework, sustained mechanical loading may be accompanied by dysregulation of NF-κB, MAPK, integrin–FAK–ERK/PI3K, and YAP signaling, which may reinforce inflammatory and matrix-degradative responses and contribute to a positive immuno-mechanical feed-forward loop linked to osteoclast activation and subchondral bone remodeling [5,13]. This conceptualization may help contextualize immune-active features of TMJOA that are not captured by structure-centered diagnostic systems and provide a theoretical basis for subsequent discussion of stage-oriented therapeutic strategies incorporating the modulation of pathological immuno-mechanical signaling [5,10,13,27,28,29,30].

TMJOA is primarily defined as a structure- and imaging-based diagnostic entity, whereas TMID is conceptualized as a mechanism-based framework that captures dynamic immune activation states and their interaction with mechanical stress over time. In this regard, TMID does not redefine the molecular components of disease, instead recontextualizing their functional relationships and temporal progression within the TMJ environment. This distinction highlights a fundamental difference in disease interpretation, shifting from static structural classification toward a dynamic, mechanism-based understanding of disease activity.

### 2.3. Anatomical and Cellular Basis of Divergent Pathophysiology

#### 2.3.1. Large Synovial Joints vs. TMJ

Large synovial joints possess spacious joint cavities and broad synovial distribution, whereas the TMJ is divided into superior and inferior compartments by an articular disc, forming a double-cavity structure in which each compartment has a relatively small absolute volume [31,32]. This anatomical distinction suggests that dilution and clearance of inflammatory mediators may be more limited in the TMJ than in large joints.

In large-joint OA, the synovial lymphatic system contributes to joint homeostasis through the removal of intra-articular cytokines and ECM-derived mediators [33]. By contrast, relatively constrained drainage capacity in the TMJ may permit the local accumulation of inflammatory mediators and facilitate immune cell retention. Such accumulation may favor repeated exposure of resident immune cells to inflammatory cues. Consequently, this microenvironment may be permissive to localized macrophage and Th17 cell reactivation and to the formation of immune feed-forward amplification loops. These features may provide an anatomical basis for the prominent local immune activation observed in TMJOA.

#### 2.3.2. Cartilage Composition: Hyaline vs. Fibrocartilage

Hyaline cartilage in large joints is rich in type II collagen and proteoglycans, which are well suited for buffering compressive loads [34]. In contrast, mandibular condylar cartilage in the TMJ has fibrocartilaginous features, with a higher proportion of type I collagen in the superficial zone and relatively low proteoglycan content [35]. Consequently, the proteoglycan-loss-driven cartilage softening typically described in large-joint OA may not manifest identically in the TMJ. Instead, such changes may appear in an attenuated or delayed manner, reflecting differences in matrix composition and load-bearing behavior [34,36].

In TMJ cartilage, lysyl oxidase-like 2 has been reported to attenuate reductions in collagen and proteoglycan expression through integrin–FAK signaling under mechanical stress, thereby contributing to ECM stability and tissue homeostasis [37]. These differences in cartilage composition and mechanobiological responsiveness may constitute a structural basis for divergent disease trajectories between TMJOA and large-joint OA.

#### 2.3.3. Retrodiscal Tissue and Double-Compartment-Mediated Amplification

The retrodiscal tissue of the TMJ is a highly vascularized and innervated fibro-elastic structure that participates in posterior disc traction and load distribution during mandibular movement [38]. In TMJ internal derangement, the retrodiscal tissue shows increased vascularity and fibrosis [39]. These magnetic resonance imaging findings vary characteristically across Wilkes stages and, in stages accompanied by limited mouth opening, may contribute to localized posterior stiffness and an imbalance in load distribution [40]. The rich vascularity of retrodiscal tissue contrasts with the adult knee meniscus, in which vascular distribution and healing capacity are largely restricted to the peripheral red zone [41].

Within the TMJ double-compartment system, fibrosis or morphological alteration in one compartment may secondarily influence the biomechanics of the adjacent compartment [32]. Increased articular loading associated with disc displacement has been reported to activate the C–C motif chemokine ligand 5 (CCL5)–C–C chemokine receptor (CCR)–protein kinase B (AKT) axis, particularly the AKT2 isoform, within the cartilage–subchondral bone interface, thereby promoting macrophage accumulation and osteoclastogenesis. These processes may enhance local inflammatory and resorptive activity and support a mechanically driven immune-mediated remodeling cascade in subchondral bone [13].

The Yes-associated protein (YAP)/transcriptional co-activator with PDZ-binding motif (TAZ) signaling has been reported to functionally couple with NF-κB, MAPK, and JAK–STAT pathways under conditions of increased ECM stiffness and inflammatory cytokine exposure, acting as a regulator of immuno-mechanical crosstalk in arthritic contexts [42]. Although similar immuno-mechanical feed-forward loops have been described in OA [20,43], the TMJ is distinguished by fibrocartilaginous condylar surfaces, narrow double joint cavities, and exposure to repetitive non-physiologic mechanical loading such as bruxism. These anatomical and biomechanical characteristics may favor reciprocal amplification between mechanical stimuli and inflammatory responses. Accordingly, the TMID framework suggests that these mechanisms may operate more prominently under joint-specific structural conditions.

## 3. Cellular Immunopathology of TMID: Macrophage–T Cell Axis

TMID is conceptualized as a pathologic continuum in which tissue damage induced by repetitive mechanical overload mobilizes innate immune activation and non-antigen-specific adaptive immune responses, and these inputs may be mutually amplified through immuno-mechanical signaling axes, potentially contributing to inflammatory amplification, tissue injury, and dysregulated bone remodeling. Within this continuum, the macrophage–T cell axis is positioned as a key immunologic axis and may represent a major driver of inflammatory amplification during the immune-active stage, thereby contributing to progression toward a fibrotic–erosive stage.

Repetitive mechanical-stress-induced tissue damage elicits innate immune responses within the synovial microenvironment and may establish a central immunologic axis in which macrophage activation and T cell recruitment drive inflammatory amplification and tissue injury [5,6,7].

### 3.1. Macrophage Polarization and Temporal Immunologic Roles

During the immune-active stage of TMID, DAMPs generated by repetitive mechanical stress promote an M1-like inflammatory program in synovial macrophages, thereby driving synovial inflammation and macrophage-associated inflammatory activity. The TLR4–myeloid differentiation primary response 88 (MyD88)–NF-κB–NLR family pyrin domain-containing 3 (NLRP3) axis has been implicated in this process [5,6,44]. Phenotypically, M1-like macrophages exhibit elevated CD80/CD86 and major histocompatibility complex class II (MHC-II) expression. They are induced by interferon-γ (IFN-γ) or TNF-α and represent a proinflammatory state functionally linked to NF-κB and JAK/STAT signaling pathways [45]. M1 polarization may induce the expression of proinflammatory cytokines, including TNF-α, IL-1β, and IL-6, as well as increased production of MMPs and ADAMTS family members such as MMP-13 and ADAMTS-5, thereby contributing to ECM and cartilage degradation. In addition, inflammatory mediator expression may be further amplified via the interleukin-17 (IL-17)–NF-κB axis [46]. In TMJOA synovial lining cells, increased inducible nitric oxide synthase (iNOS) expression has been linked to nitrosative stress and apoptosis, potentially contributing to the persistence of synovitis [47,48]. Concurrently, TNF-α- and IL-1β-dominant inflammatory milieus induce C–C motif chemokine ligands, including CCL20 and CCL5, thereby promoting recruitment of C–C motif chemokine receptor 6 (CCR6)^+^ T cells and sustaining inflammatory amplification [7]. The macrophage–Th17–RANKL axis described in RA links inflammatory cytokine production to osteoclast activation, and it provides a conceptual reference for a potentially analogous immune–bone coupling axis operating in TMJOA lesions [49].

Conversely, during chronic stages or resolution phases, macrophages may transition from an M1 toward an alternatively activated M2 (anti-inflammatory) phenotype in accordance with changes in local cytokine composition and increased anti-inflammatory signals. Interleukin-4/interleukin-13–signal transducer and activator of transcription 6 (STAT6) and interleukin-10 (IL-10)–signal transducer and activator of transcription 3 (STAT3) signaling promote M2 polarization [50], and therapeutic stimuli such as S-propargyl-cysteine have been reported to attenuate M1 programs and facilitate M1–M2 switching through JAK–STAT inhibition [10]. M2 macrophages are characterized by the secretion of IL-10 and TGF-β1. They may also induce CD4^+^ T cell differentiation toward regulatory T cells (Tregs) via TGF-β–small mothers against decapentaplegic (Smad) signaling [51]. In parallel, M2 macrophages may facilitate local Treg recruitment through C-C motif chemokine ligand 22 (CCL22) and the C-C motif chemokine ligand 1–C-C motif chemokine receptor 8 (CCL1–CCR8) axis. In addition, the dominance of the arginase–polyamine axis establishes a metabolic environment favorable for tissue repair and remodeling. IL-10 restricts inflammatory gene expression through STAT3-dependent mechanisms and NF-κB suppression [52], and it has been reported to inhibit IFN-γ-induced iNOS expression and nitric oxide (NO) production [53].

Subsets of M2-associated macrophages further promote vascular stabilization and tissue regeneration by secreting regulatory factors such as platelet-derived growth factor-BB, tissue inhibitor of metalloproteinases 3, and MMP-9, as well as growth factors such as insulin-like growth factor 1, transforming growth factor-β1, and vascular endothelial growth factor A (VEGF-A) [54,55]. M2-associated IL-10 and TGF-β1 may additionally modulate local nociceptive signaling pathways, potentially contributing to attenuation of pain-related sensitization [56,57].

Collectively, the temporal switching and functional plasticity of macrophage polarization provide an interpretive framework for understanding coordinated immune regulation, tissue remodeling, and pain modulation within TMID pathophysiology.

### 3.2. T Cell Subset Dynamics in TMID Stages: Th1–Th17–Treg Axis

In inflammatory TMJOA synovium, activation of the CXC chemokine ligand 9–11–CXC chemokine receptor 3 (CXCL9–11–CXCR3) and CCL20–CCR6 axes has been suggested to contribute to localized T cell recruitment. Combined stimulation by Th1-associated IFN-γ and TNF-α has been reported to markedly amplify the expression of these chemokines in synovial fibroblasts [58]. Synovial fluid-derived cells from TMJOA patients exhibit increased expression of Th1/Th17-related cytokines, including IL-1β, IL-17, and RANKL, together with reduced interleukin-4 and TGF-β1 levels, indicating an immune bias favoring osteoclastogenic mediators [15].

IL-17 produced by Th17 cells reinforces inflammatory microenvironments enriched in TNF-α and IL-1β and increases RANKL expression in osteoblasts and mesenchymal cells, which in turn promotes osteoclastogenesis and bone resorption [59]. In periodontal ligament cells, IL-17 has been reported to decrease osteoprotegerin (OPG) expression through MAPK, AKT, and NF-κB signaling, leading to an increased RANKL/OPG ratio and contributing to the formation of a pro-resorptive microenvironment [60].

During chronic or resolving phases of TMID, changes in the local cytokine milieu may favor a shift in macrophage polarization from M1 toward M2 [50], and the resulting TGF-β–Smad signaling environment may promote differentiation of CD4^+^ T cells into Tregs [51]. This coordinated transition is accompanied by attenuation of Th1/Th17-dominant immune activity and establishment of an M2/Treg-favoring regulatory milieu [50,51,52]. In parallel, local Treg recruitment may be facilitated through the CCL1–CCR8 and CCL22–C-C motif chemokine receptor 4 (CCR4) axes [52]. In osteoarthritis contexts, Tregs suppress Th1/Th17-driven inflammation primarily through IL-10- and TGF-β-mediated mechanisms [61,62]. Impaired Treg function or Th17/Treg imbalance has been associated with persistent inflammation, cartilage degeneration, and bone destruction [61], whereas restoration of Treg activity may restrain RANKL-dependent osteoclast differentiation and reduce bone resorption [62].

During regenerative or healing phases, Treg-derived IL-10 and amphiregulin support tissue repair and can reprogram macrophages toward pro-healing states, contributing to inflammation resolution and IL-10-mediated analgesic effects; moreover, reduced Treg abundance or function may be associated with pain and functional impairment in osteoarthritis [62].

Collectively, TMJOA appears to be characterized by recurrent immune-active states dominated by Th1 and Th17 responses, whereas findings from osteoarthritis and broader immunologic studies suggest the potential for transition toward Treg-centered resolution and regenerative phases. Such immune phase transitions reflect temporal dynamics of immune responses influenced by mechanical stimuli and provide a pathophysiologic basis for the TMID framework.

**Figure 1:**
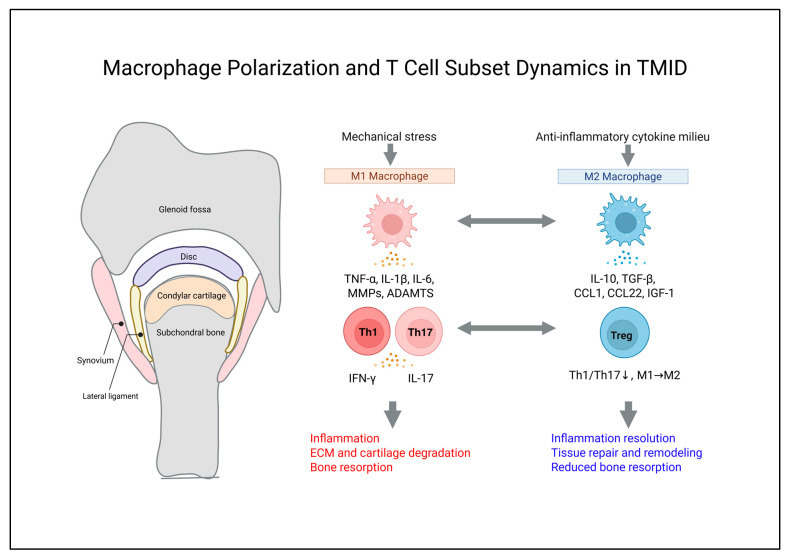
Macrophage polarization and T cell subset dynamics in temporomandibular immunologic disease (TMID). This schematic summarizes representative literature-based immune relationships proposed in temporomandibular joint (TMJ) pathology. Mechanical stress and inflammatory milieus are associated with classically activated M1 (pro-inflammatory) macrophage polarization and T helper 1 (Th1) and T helper 17 (Th17) activation, which may facilitate inflammatory responses, extracellular matrix (ECM) and cartilage degradation, and subchondral bone resorption. In contrast, anti-inflammatory cytokine milieus favor alternatively activated M2 (anti-inflammatory) macrophage polarization and regulatory T cell (Treg) responses, which collectively suppress inflammatory cascades and limit ECM/cartilage breakdown and bone resorption, thereby supporting restoration of local immune homeostasis. This schematic represents a conceptual synthesis based on representative findings from TMJ osteoarthritis and immunologic studies [5,6,7,50,58,59,61]. Created in BioRender. KIM, H. (2026) https://BioRender.com/i1k8xs9 (accessed on 1 March 2026).

The macrophage polarization and T cell subset dynamics in TMID are illustrated in Figure 1.

## 4. Integration of Immune and Mechanical Signaling and Tissue Remodeling in TMJ Pathology

### 4.1. Immune-Mediated Inflammatory and Mechanotransductive Pathways in TMID

**Figure 2:**
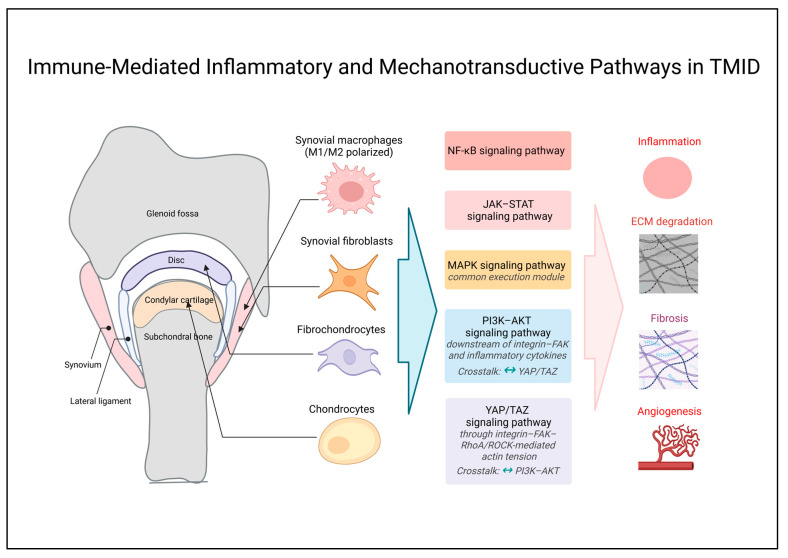
Immune-mediated inflammatory and mechanotransductive pathways in temporomandibular immunologic disease (TMID). This schematic summarizes representative literature-based signaling relationships reported in temporomandibular joint (TMJ) pathology. Inflammatory signaling pathways centered on nuclear factor-κB (NF-κB) and Janus kinase–signal transducer and activator of transcription (JAK–STAT) are activated across TMJ-resident cells, with mitogen-activated protein kinase (MAPK) functioning as a common downstream execution module integrating inflammatory and mechanical stimuli [9,10,44,58,63,64]. Mechanotransductive responses mediated by integrin–focal adhesion kinase (FAK) and phosphoinositide 3-kinase (PI3K)–protein kinase B (AKT) under mechanical loading, along with their interaction with Yes-associated protein (YAP)/transcriptional co-activator with PDZ-binding motif (TAZ) signaling, are supported by TMJ-specific and joint-related studies [5,65,66,67,68]. These pathways collectively contribute to key pathological outputs in TMID, including inflammation, extracellular matrix (ECM) degradation, fibrosis, and angiogenesis [11,63,69,70]. Created in BioRender. KIM, H. (2026) https://BioRender.com/i1k8xs9 (accessed on 1 March 2026).

In TMID, mechanical overload and inflammatory cytokines may amplify ECM degradation, fibrosis, and pathological angiogenesis through multiple signaling axes. In this section, inflammatory immune signaling centered on NF-κB and JAK–STAT is outlined, with MAPK often functioning as an execution module, following which convergence with mechanotransductive pathways is discussed, represented by integrin–FAK, PI3K–AKT, and YAP/TAZ. Certain mechanisms, particularly the YAP/TAZ–MRTF axis, are presented as conceptual extensions for TMJ pathophysiology based on evidence from non-TMJ systems. These signaling pathways should not be interpreted solely as primary drivers of disease initiation but, rather, as context-dependent processes that may function both as downstream responses and as secondary amplifiers within a sustained immuno-mechanical feedback loop. Accordingly, the relationships described in this framework are better understood as associative and dynamic interactions rather than definitive causal hierarchies. The immune-mediated inflammatory and mechanotransductive pathways in TMID are illustrated in Figure 2.

To improve the interpretability of the signaling network presented in Figure 2, a structured summary of key mechanical drivers, immune mediators, convergent signaling pathways, and their downstream outputs across the TMID framework is provided in Table 3 as a complementary overview to the schematic representation.

Accordingly, the following sections summarize representative immunologic and mechanotransductive signaling axes implicated in TMJ pathology, emphasizing their functional convergence within the proposed TMID framework.

#### 4.1.1. NF-κB Signaling Pathway

The NF-κB pathway serves as a central transcriptional axis integrating inflammatory and stress responses during active TMID stages and is activated across major TMJ cell populations, including synovial fibroblasts [58], synovial macrophages [44], condylar chondrocytes [63], disc cells [71,72], synovial mesenchymal stem cells (MSCs) [7], and osteoblasts [73]. Accordingly, NF-κB activity in TMJOA functions as an upstream regulator coordinating expression of inflammatory cytokines and matrix-degrading enzymes across synovium, cartilage, and subchondral bone [9].

Principal upstream triggers include TLR2/4 and tumor necrosis factor receptor (TNFR) stimulation, which drive transcriptional programs through a canonical cascade involving MyD88, IκB kinase (IKK) complex activation, cytoplasmic inhibitor of κB alpha (IκBα) phosphorylation and degradation, and subsequent NF-κB nuclear activation [73]. In TMJ condylar chondrocytes, IL-1β activates NF-κB signaling and—together with wingless-type MMTV integration site family member 5A (Wnt5a)-associated signaling—is linked to increased matrix-degrading enzymes and cartilage degeneration [69]. Enhanced TLR4–MyD88–NF-κB signaling is observed in TMJOA synovium and condylar cartilage and is associated with synovitis, cartilage degeneration, matrix loss, and increased subchondral bone destruction with osteoclast activation [44,69].

NF-κB functions as a key regulator of catabolic gene programs in condylar chondrocytes. Osteopontin promotes nuclear translocation of NF-κB p65 (RelA) and upregulates MMP-1/3/9/13 [8,74]. Similarly, periostin induces NF-κB activation accompanied by IκBα phosphorylation and degradation and p65 nuclear translocation, with associated increases in ADAMTS-4/5 and MMP-13, thereby linking periostin signaling to catabolic remodeling and resorption [75]. High-intensity cyclic tensile strain increases NF-κB phosphorylation and elevates cyclooxygenase-2 (COX-2), prostaglandin E_2_ (PGE_2_), MMP-1/3/9, and ADAMTS-5 while reducing collagen type II alpha 1 chain (*COL2A1*) and aggrecan expression [63]. In IL-1β-induced TMJOA, TLR2–NF-κB p65 coupling mediates TNF-α, COX-2, and MMP-3/13 responses, while mechanical pressure loading has also been reported to upregulate NF-κB p65 through TLR2-independent mechanisms [76].

NF-κB is further linked to pathological angiogenesis. IL-1β–NF-κB signaling is associated with increased VEGF expression [69] and, in perforated TMJ disc cells, VEGF upregulation is accompanied by reduced chondromodulin-1 (ChM-1) and thrombospondin-1 (TSP-1), reinforcing pro-angiogenic tendencies [71]. In TMJ disc-derived fibrocartilaginous cells, NF-κB activation is associated with disturbed collagen synthesis–degradation homeostasis, aberrant collagen remodeling, and deterioration of mechanical properties [72].

In TMJ synovial fibroblasts, TNF-α-induced NF-κB activation is associated with increased interleukin-8 and CXCL10 expression, which may contribute to inflammatory responses and immune cell recruitment within the synovial microenvironment [58]. In fibroblast-like synoviocytes, IL-1β and TNF-α activate ERK1/2, p38 mitogen-activated protein kinase (p38), c-Jun N-terminal kinase (JNK), and NF-κB pathways to mediate CCL20 production, which is associated with the recruitment of CCR6^+^ immune cells [77]. High mobility group box 1 (HMGB1) induces the phosphorylation and nuclear accumulation of NF-κB p65, increasing MMP-13, ADAMTS-5, IL-1β, and IL-6 expression and exacerbating cartilage and subchondral bone damage [78]. In macrophage-dominant inflammatory milieus, increased reactive oxygen species (ROS) associated with TLR4–MyD88–NF-κB–NLRP3 signaling amplify inflammatory cytokine release and contribute to joint-wide inflammatory amplification [44].

Collectively, NF-κB signaling may act as a major integrative node linking mechanical overloading and inflammatory cues in TMJ lesions and provides a mechanistic basis for the immuno-mechanical signaling axis proposed in TMID.

The NF-κB-mediated inflammatory and catabolic signaling in TMID is illustrated in Supplementary Figure S1.

#### 4.1.2. JAK–STAT Signaling Pathway

JAK–STAT signaling is activated across major TMJ cell populations by IFN-γ, IL-6, and TNF-α through their cognate cytokine receptors, as well as by leptin [10,14,58]. This pathway functions as a core immune signaling axis that drives inflammatory transcriptional programs via JAK2 activation and phosphorylation of signal transducer and activator of transcription 1 and 3 (STAT1/3), followed by STAT1/3 dimerization and nuclear transcriptional activation.

In TMJOA-related macrophage models, JAK2/STAT3 activation has been reported to increase iNOS, IL-6, and TNF-α expression, thereby promoting M1 polarization [10].

Within inflammatory TMJ microenvironments, T cell-derived interferon-γ induces STAT1 phosphorylation in synovial fibroblasts and, together with TNF-α, increases CXCL9–11 expression [58]. STAT1 activity is associated with increased MMP-3/13 expression, potentially contributing to ECM degradation and deterioration of the cartilage–subchondral bone architecture [79].

Elevated leptin in synovial fluid from TMJOA patients activates JAK2/STAT3 via leptin receptor long isoform (Ob-Rb) in synovial fibroblasts and increases IL-6 expression [14]. This response is linked to the formation of an inflammatory TMJ microenvironment [80], ADAMTS-4/5-associated cartilage ECM degradation, and VEGF-associated angiogenic responses [69].

In addition, increased C–X3–C motif chemokine ligand 1 (CX3CL1) associated with JAK–STAT activity has been suggested to contribute to apoptosis-related signaling and osteoclast precursor chemotaxis [81].

Collectively, the JAK–STAT pathway, together with NF-κB and MAPK, constitutes a core inflammatory immuno-mechanical signaling axis in TMID and participates in integrated regulation of inflammatory pathologic responses in TMJ lesions. The JAK–STAT signaling and TMJ transcriptional outputs in TMID are illustrated in Supplementary Figure S2.

#### 4.1.3. MAPK Signaling Pathway

MAPK signaling comprises a kinase cascade involving mitogen-activated protein kinase kinase kinases (MAP3Ks) (Raf, MEKK, transforming growth factor-β–activated kinase 1 (TAK1), and ASK1), mitogen-activated protein kinase kinases (MAP2Ks) (MEK1/2, MKK3/6, and MKK4/7), and MAPKs (ERK1/2, JNK, and p38), which is activated by extracellular cytokines, stress signals, and receptor stimulation. This cascade functions as a common execution module that responds to both inflammatory and mechanical stimuli in TMJ lesions [64,82]. Activated ERK1/2, JNK, and p38 phosphorylate transcription factors, including c-Fos, c-Jun, and activating transcription factor 2 (ATF2), subsequently activate activator protein-1 (AP-1)-dependent transcriptional programs. These outputs are characterized by increased MMP-1/3/13, ADAMTS-5, VEGF, CX3CL1, and Runt-related transcription factor 2 (*RUNX2*), together with reduced *SOX9* and *COL2A1* expression [5,64,81,83].

MAPK activation has been reported in multiple TMJ-related cell populations, including fibroblast-like synoviocytes [11,69], condylar chondrocytes [9,69], disc cells [71], synovial MSCs [69], and subchondral bone marrow stromal cells [84]. In TMJ synovial fibroblast-like cells, IL-1β and TNF-α activate ERK1/2, p38, and JNK, mediating CCL20 production and potentially contributing to recruitment of CCR6^+^ immune cells [77]. Elevated leptin in synovial fluid from TMJOA patients activates p38 MAPK via Ob-Rb and increases IL-6 expression [14]. This response is linked to ADAMTS-4/5-mediated cartilage ECM degradation and ERK1/2–estrogen-related receptor gamma (ERRγ)–VEGF-mediated angiogenesis [69].

In condylar chondrocytes, IL-1β stimulation activates the JNK/c-Jun pathway, suppressing *SOX9* and *COL2A1* expression [83], while p38 MAPK activation increases CX3CL1, promoting osteoclast precursor chemotaxis and contributing to focal bone resorption [81]. Excessive mechanical loading induces ERK1/2 and PI3K phosphorylation through the integrin–FAK axis and elicits catabolic responses characterized by increased MMP-1/3/13 and ADAMTS-5, while reducing *COL2A1* expression [5]. In TMJ disc fibrochondrocytes, MEK–ERK1/2 and p38 MAPK activation mediates MMP upregulation and catabolic remodeling [85] and, in perforated disc cells, IL-1β induces parallel activation of p38 MAPK and NF-κB, promoting angiogenic factor imbalance, endothelial migration, and tube formation [71]. In subchondral bone, mechanical overload-induced Wnt5a/receptor tyrosine kinase-like orphan receptor 2 (Ror2)–JNK–Ca^2+^/nuclear factor of activated T cells (NFAT) signaling increases CXC chemokine ligand 12 (CXCL12) and RANKL expression, facilitating osteoclast precursor migration and differentiation, while simultaneously enhancing *RUNX2* expression and mineralization in bone marrow-derived MSCs [84]. Collectively, the MAPK pathway functions as an execution module responsive to both inflammatory and mechanical cues in TMJ lesions. The MAPK–AP-1 signaling and TMJ transcriptional outputs in TMID are illustrated in Supplementary Figure S3.

#### 4.1.4. Integrin–FAK–PI3K–AKT Mechanotransduction and MAPK Signaling

Mechanical overloading activates FAK through integrins and subsequently activates MAPK (ERK1/2, JNK, p38) and PI3K–AKT signaling pathways in parallel. Consequently, the integrin–FAK–MAPK and integrin–FAK–PI3K–AKT axes function as a mechanotransduction signaling hub that integrates mechanical inputs with inflammatory cytokine cues in TMJ lesions [5,65,66,86]. In condylar chondrocytes, physiologic mechanical stimulation contributes to cell survival and suppression of apoptosis through integrin–FAK-mediated ERK1/2 and PI3K phosphorylation [65]. In contrast, excessive loading induces FAK phosphorylation, together with the hyperactivation of MAPK (ERK1/2, JNK, and p38) and PI3K–AKT, leading to increased MMPs and ADAMTS and reduced *COL2A1* and aggrecan, with consequent ECM degradation [5,86].

Downstream MAPK signaling under FAK enhances catabolic transcriptional programs through AP-1 activation [5]. In parallel, the PI3K–AKT–mechanistic target of rapamycin (mTOR) axis mediates reduced autophagy and increased chondrocyte apoptosis [69] and, under hypoxic conditions, hypoxia-inducible factor-1 alpha (HIF-1α) stabilization with increased VEGF expression may contribute to angiogenesis and degenerative changes [86]. Mechanical overload also activates mechanosensitive Ca^2+^ channels such as transient receptor potential vanilloid 4 (TRPV4) and Piezo1, inducing Ca^2+^ influx. In addition, transient receptor potential vanilloid 5 (TRPV5)-mediated Ca^2+^ entry has been reported to activate Ca^2+^/calmodulin-dependent protein kinase II (CaMKII)–MAPK and AKT/mTOR signaling and promotes chondrocyte apoptosis. TRPV4 activation further amplifies inflammatory cytokine and MMP expression, accelerating ECM degradation [5,87].

FAK–MAPK signaling activated by mechanical stimulation intersects downstream with the interleukin-1 receptor 1 (IL-1R1)–MyD88–interleukin-1 receptor-associated kinase (IRAK)–TNF receptor-associated factor 6 (TRAF6)–TAK1 inflammatory axis, and the TAK1-mediated amplification of ERK, JNK, and p38 may reinforce catabolic transcriptional responses in TMJ tissues [88]. In synovial fibroblast-like synoviocytes, mechanical loading or TNF-α stimulation activates PI3K–AKT and increases cadherin-11, vascular endothelial growth factor D (VEGF-D), and fibroblast growth factor 2 (FGF-2) expression, thereby enhancing synovial angiogenic capacity [66]. Within inflammatory TMJOA milieus, leptin–Ob-Rb signaling activates p38 MAPK and PI3K–AKT and increases IL-6 expression [14].

In OA chondrocyte models, IL-1β activates the PI3K/AKT–NF-κB axis and increases COX-2, iNOS, PGE_2_, TNF-α, IL-6, MMP-3/13, and ADAMTS-5 expression [89]. In non-TMJ systems, the PI3K–AKT axis promotes nuclear accumulation of YAP/TAZ and increases cysteine-rich angiogenic inducer 61 (*CYR61*) expression. In addition, YAP/TAZ–TEA domain family member (TEAD) interactions and TAZ–Smad3 coupling drive the expression of alpha-smooth muscle actin (α-SMA), connective tissue growth factor (CTGF), and collagen I, thereby promoting fibrotic transcriptional programs [67,90]. Thus, PI3K–AKT signaling functionally intersects with YAP/TAZ mechanotransduction and contributes to NF-κB activation, linking mechanical sensing to inflammatory and fibrotic transcriptional outputs.

Collectively, these signaling axes provide a molecular basis through which mechanical overload and inflammatory stimuli are integrated into degenerative tissue responses in TMJ lesions and may represent core execution modules in TMID pathophysiology. The integrin–FAK–PI3K–AKT mechanotransduction and downstream pathogenic signaling in TMID are illustrated in Supplementary Figure S4.

#### 4.1.5. YAP/TAZ Signaling Pathway

Evidence of YAP/TAZ-mediated mechanotransduction in joint degeneration has mainly been derived from non-TMJ systems, and direct validation in TMJ tissues remains limited. Accordingly, YAP/TAZ signaling is discussed here not as a definitive pathway in TMID but as a conceptual framework for understanding how mechanical stress, ECM stiffening, and inflammatory or hypoxic microenvironments may converge toward degenerative tissue responses.

Mechanical stimulation or increased ECM stiffness suppresses the Hippo kinase cascade (MST1/2–LATS1/2) through integrin–FAK–Src activation and RhoA–ROCK–actin stress fiber formation, leading to YAP/TAZ dephosphorylation and nuclear translocation [90,91]. The PI3K–AKT axis has also been reported to promote nuclear accumulation of YAP/TAZ and increase *CYR61* transcription [67]. Nuclear YAP/TAZ associates with TEAD to induce the expression of fibrosis-related genes, including α-SMA, CTGF, and collagen type I. In addition, TAZ–Smad3 interactions further reinforce myofibroblast differentiation [90]. Therefore, YAP/TAZ activity may establish a feed-forward loop coupled to ECM stiffening [90].

YAP/TAZ has been linked to crosstalk with inflammatory signaling, including NF-κB, and to functional coupling with the TGF-β–Smad3, PI3K/AKT, and β-catenin pathways. These interactions are associated with matrix-degrading programs, ECM degradation, fibrotic responses, angiogenesis, and Th17/Treg imbalance [92]. In synovium, YAP/TAZ-related signaling networks involving IL-6–YAP, TGF-β–Smad3, and mTOR are associated with invasive phenotypes, synovial lining hyperplasia, immune cell infiltration, and fibrosis [92]. YAP has been implicated in PI3K/AKT-dependent angiogenic programs, whereas TAZ has been associated with enhanced Th17 differentiation and suppression of Treg differentiation, thereby contributing to Th17/Treg imbalance [92].

In subchondral bone and MSCs, YAP/TAZ has been reported to interact with Wnt/β-catenin and TGF-β signaling and to regulate *RUNX2* expression, osteogenic differentiation, and bone remodeling [92]. In the TMJ context, YAP has been reported to participate in bony ankylosis formation through MSC-dependent processes [68].

Within TMJ tissues, excessive mechanical loading increases integrin–FAK activation in condylar chondrocytes together with MAPK, NF-κB, and AKT signaling, leading to increased TNF-α, IL-1β, COX-2, and MMP-3/13 expression and degradation of type II collagen and aggrecan [93]. Hypoxia stabilizes HIF-1α and increases VEGF expression, facilitating angiogenesis at the osteochondral junction [70]. Mechanical loading and inflammatory cues activate the β-catenin–RUNX2 axis, leading to increased MMP-13 and ADAMTS-4/5 expression and pathological remodeling of the cartilage–bone interface [94,95]. In disc cells, IL-1β, IL-6, TNF-α, and hypoxia induce VEGF expression and are associated with disc degeneration [96], while symptomatic internal derangement is accompanied by increased synovial VEGF and angiogenesis linked to persistent synovitis [97]. In TMJ fibrocartilage, excessive loading is associated with disrupted FAK signaling and altered expression of ECM remodeling factors, including MMP-13 and TGF-β, suggesting involvement of an integrin–FAK–YAP axis in fibro-degenerative remodeling [12]. In addition, inhibition of ROCK–actin–MRTF signaling in TMJ-derived fibroblast-like synoviocytes reduces α-SMA and collagen I expression, supporting a role for the RhoA–ROCK–actin axis in TMJ fibrosis and suggesting a potential functional link to YAP/TAZ signaling [11].

The YAP/TAZ-mediated mechanotransduction and TMJ-associated pathological outcomes in TMID are illustrated in Supplementary Figure S5.

### 4.2. Subchondral Bone Remodeling in TMID

In late-stage TMID, subchondral bone exhibits pathological remodeling characterized by flattening, erosion, sclerosis, osteophyte formation, subchondral cysts, and bone loss [98,99]. These changes are increasingly viewed not simply as secondary degenerative consequences but as dynamic structural manifestations of immuno-mechanical dysregulation within the osteochondral unit. Within the TMID framework, these structural phenotypes are interpreted as downstream outputs of sustained immuno-mechanical signaling convergence at the cartilage–subchondral bone interface. Repetitive mechanical stress, including bruxism, increases condylar loading through disc displacement, joint space narrowing, and elevated articular surface friction [100]. In addition, persistent overload after discectomy is associated with cartilage thinning and proteoglycan loss [76]. Such excessive mechanical burden promotes microfractures and microcracks formation at the osteochondral interface [5], while synovial fluid entry through fissured cartilage may lead to cavity formation and subsequent release of DAMPs [101,102].

Released DAMPs activate TLR2–NF-κB signaling, as well as HMGB1-mediated TLR2/4–receptor, for advanced glycation end products (RAGE)–NF-κB pathways, resulting in increased TNF-α, COX-2, MMP-13, ADAMTS-5, IL-1β, and IL-6 expression. These changes promote cartilage matrix degradation and subchondral bone destruction [5,76,78]. Concurrently, enhanced TLR4–MyD88–NF-κB and MAPK activity within the cartilage–subchondral bone unit links synovial inflammation, cartilage degeneration, chondrocyte pyroptosis, and subchondral bone destruction into an inflammatory–catabolic loop [9,44]. These observations support the concept that cartilage and subchondral bone function as an integrated pathobiological unit in TMID, rather than as independent tissue compartments.

Chondrocyte apoptosis together with p38 MAPK–CX3CL1 upregulation promotes recruitment of bone marrow-derived macrophages and monocytes [81]. Infiltrating immune cells, together with RANKL/OPG imbalance, elevated synovial TNF-α, IL-1β, IL-6, and IL-17A, increased MMP-13, and reduced IL-10 in M1-polarized macrophages, and ROS accumulation collectively amplify osteoclastogenesis and pathological bone resorption [16,44,81,103]. Moreover, Wnt5a/Ror2–Ca^2+^/NFAT signaling increases CXCL12 and RANKL expression, further facilitating osteoclast precursor migration and differentiation [5,84]. Together, these mechanisms indicate that immune-driven osteoclast activation represents a major contributor to subchondral bone loss in TMID.

Collectively, these findings exemplify an osteoimmunologic microenvironment in which macrophage–osteoclast coupling constitutes a central driver of immune-mediated subchondral bone resorption.

Aberrant loading-induced fibroblast growth factor receptor 1–mTOR signaling increases MMP-13, ADAMTS-5, collagen type X alpha 1 chain (*COL10A1*), and *RUNX2* expression while reducing aggrecan expression. This signaling is associated with heightened catabolic activity, chondrocyte hypertrophy, and suppression of autophagy [104]. These changes may compromise the mechanical buffering capacity of the osteochondral junction and contribute to the deterioration of subchondral bone microarchitecture. In parallel, chondrocyte hypertrophy and phenotypic transition of subsets of hypertrophic chondrocytes toward osteoblast- or osteocyte-like cells contribute to subchondral bone formation [8,95]. Activation of the β-catenin–RUNX2 axis further reinforces cartilage–bone crosstalk-driven catabolic remodeling linked to increased MMP-13, ADAMTS-5, and *COL10A1* expression [94].

During osteoarthritis progression, increased osteoblast TGF-β1 expression, elevated osteoclast activity, and increased thickness and stiffness of the osteochondral interface have been reported [8,73]. Osteoblast-derived VEGF promotes neovascularization, cartilage ossification, mineral deposition, and osteophyte formation at the osteochondral junction [69,73]. Multiple converging pathways, including VEGF–vascular endothelial growth factor receptor 2 (VEGFR2)–Delta-like ligand 4 (Dll4)–Notch, HIF-1α–VEGF, HMGB1–JNK/ERK, and IL-6–ERK1/2–ERRγ, appear to drive aberrant angiogenic–ossification remodeling [69]. Consistent with these processes, reductions in bone mineral density, decreased bone volume fraction, reduced trabecular thickness, and increased trabecular separation reflect progressive subchondral bone deterioration [105,106]. Furthermore, sympathetic activation via α2A- and β2-adrenergic receptor–ERK1/2, AKT, and protein kinase A (PKA) signaling increases MMP-3/13 and RANKL expression, thereby exacerbating osteochondral complex degradation and subchondral bone loss [5,106].

Radiographically, early stages show flattening followed by erosion, which are associated with early degenerative change, active or unstable joint status, and pain [98,107,108,109]. Progressive stages exhibit sclerosis characterized by a dense subchondral bone plate and marginal osteophytes [99,107,110], whereas late-stage disease is typified by osteolytic subchondral cysts associated with disc displacement without reduction (DDwoR) [101,102,111]. Collectively, these imaging features may be interpreted as structural phenotypes reflecting stage-dependent activation of the immuno-mechanical signaling axes proposed in TMID.

**Figure 3:**
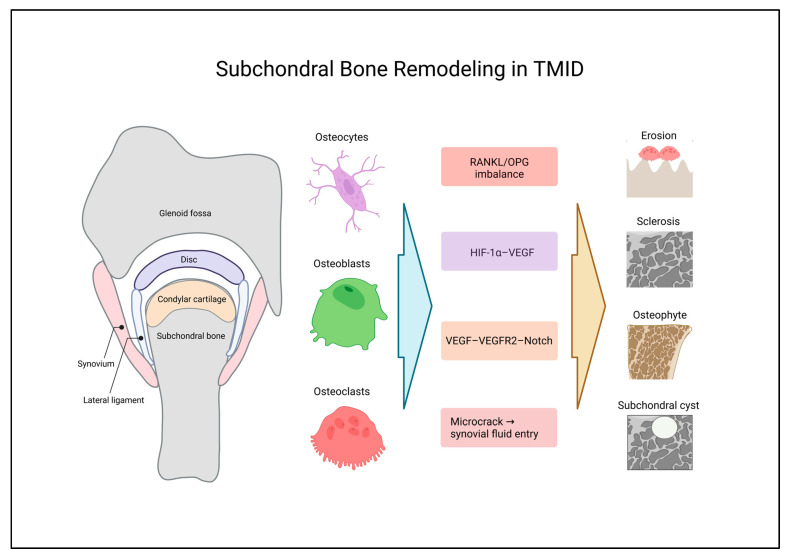
Subchondral bone remodeling in temporomandibular immunologic disease (TMID). This schematic summarizes key literature-based mechanisms underlying osteoimmunologic remodeling of the subchondral bone in temporomandibular joint (TMJ) pathology. Receptor activator of nuclear factor κB ligand (RANKL)/osteoprotegerin (OPG) imbalance-associated osteoclast activation mediates resorptive remodeling of the subchondral bone. Osteoblast responses linked to hypoxia-inducible factor 1-alpha (HIF-1α)–vascular endothelial growth factor (VEGF) signaling contribute to sclerotic remodeling. Periosteal osteoblast activity associated with VEGF–vascular endothelial growth factor receptor 2 (VEGFR2)–Notch signaling is implicated in osteophyte formation. In parallel, microstructural damage involving osteocytes and osteoclast activity contributes to subchondral cyst formation. These features collectively reflect subchondral bone remodeling driven by mechanical loading and inflammatory cues in TMID [5,16,69,73,81,101]. Created in BioRender. KIM, H. (2026) https://BioRender.com/i1k8xs9 (accessed on 1 March 2026).

Subchondral bone remodeling in TMID is illustrated in Figure 3.

## 5. Therapeutic Implications and Future Perspectives

### 5.1. Limitations of Conventional Therapies in TMID

Current treatment strategies can contribute to pain control and functional improvement in patients across the TMD/TMJOA spectrum. However, available evidence remains limited regarding whether these approaches achieve stage-specific reprogramming of the core immuno-mechanical signaling axes proposed to underlie TMID, including the NF-κB, MAPK, STAT1, integrin–FAK, PI3K–AKT, and Wnt/β-catenin pathways [5,9,27,28]. In this context, conventional care often functions primarily as symptomatic management or biomechanical unloading, with the extent to which these approaches modify the upstream immuno-mechanical circuitry that sustains catabolic and inflammatory transcriptional programs remaining uncertain [5,9,28].

Nonsteroidal anti-inflammatory drugs alleviate pain and acute inflammatory signs primarily through COX-2 inhibition [27]. Nonetheless, COX-independent activation of immuno-mechanical signaling can persist [9], and key upstream inputs—such as DAMP-initiated PRR signaling and mechanotransductive integration through integrin–FAK and PI3K–AKT—may remain active despite short-term symptomatic relief [5,9]. Similarly, although splint therapy and physical modalities can reduce joint loading and improve clinical symptoms, whether they substantially modify integrin–FAK–ERK1/2- and PI3K–AKT-based mechanosensing programs has not been clearly established. It also remains unclear whether these approaches disrupt the catabolic and inflammatory feed-forward loops coupled to NF-κB, MAPK, and Wnt/β-catenin signaling [5,28,112]. This gap is particularly relevant within the TMID framing, in which therapeutic timing and disease activity are conceptualized around dynamic immune activation and remodeling phenotypes rather than solely around structural descriptors.

Surgical interventions may improve function in selected settings [113]; however, procedure-associated tissue injury could theoretically induce DAMP release and secondary activation of TLR2/4 signaling [4]. In preclinical TMJOA models, activation of the TLR4–MyD88–NF-κB–NLRP3 axis has been associated with chondrocyte pyroptosis, increased ADAMTS-5 and MMP-13 expression, and amplification of M1 macrophage-mediated inflammatory responses [44]. Although these observations do not indicate that surgical intervention uniformly worsens immunopathology, they do suggest that peri-procedural tissue injury may, at least in principle, intersect with DAMP–TLR pathways that are central to the proposed immuno-mechanical framework [4,44]. Despite this, mechanistic considerations of such immuno-mechanical risk plausibility are rarely addressed explicitly in the surgical literature, which has largely focused on clinical outcomes and structural endpoints [113].

Adjunctive approaches targeting pain processing and muscle-related symptoms, including cognitive behavioral therapy and botulinum toxin injections, have demonstrated analgesic and myofascial symptom-relieving effects [114,115,116]. However, these modalities do not necessarily reprogram the joint-level immuno-mechanical signaling axes emphasized in TMID. In addition, repeated administration of botulinum toxin has been associated with a risk of muscle atrophy [117]. Taken together, these limitations suggest that conventional therapies, while clinically valuable, may incompletely address the hypothesized upstream drivers and sustaining loops of TMID across stages [5,9,28].

Accordingly, TMID-oriented management may need to extend beyond symptom-oriented strategies toward pathophysiology-based approaches. Such approaches would ideally combine mechanical load modulation with immunomodulatory and regenerative interventions targeting immuno-mechanical signaling networks in a stage-matched manner aligned with disease activity and remodeling phenotypes [5,9,27,28].

Importantly, in light of the limited capacity of conventional therapies to modulate upstream immuno-mechanical signaling pathways, and in contrast to conventional Diagnostic Criteria for Temporomandibular Disorders (DC/TMD)-based approaches that primarily guide management based on structural findings and symptom classification, TMID is not intended to replace current DC/TMD-based clinical decision-making or to provide an immediate treatment algorithm. Rather, it introduces a mechanism-based interpretive layer suggesting that patients with similar structural TMJOA findings may differ in underlying disease activity. These differences may include immune-active, mechanically dominant, or fibrotic–remodeling states. For example, such patients may be stratified into immune-active versus mechanically dominant phenotypes, which could guide the differential application of anti-inflammatory interventions in immune-active states versus load-modifying or biomechanical approaches in mechanically dominant conditions.

This framework supports a shift toward stage-aware and mechanism-informed therapeutic strategies, in which treatment selection and timing are guided not only by structural findings but also by inflammatory activity, immune status, and remodeling dynamics. In this context, TMID provides a conceptual basis for future patient stratification and targeted therapeutic approaches integrating load modulation, immunomodulation, and regenerative interventions.

### 5.2. Emerging Immuno-Regenerative Strategies

Recent studies have proposed multiple immuno-regenerative strategies for TMID that aim to reprogram inflammatory microenvironments while promoting tissue repair. This section focuses on emerging mechanistically oriented approaches and excludes conventional intra-articular biologics such as platelet-rich plasma or hyaluronic acid. Given that most supporting evidence remains preclinical and TMJ-specific validation is limited, these strategies are discussed as mechanistic exemplars rather than clinical recommendations. Key translational challenges include the development of TMJ-focused disease models, standardized formulations, optimized dosing regimens and delivery strategies, and long-term safety assessment.

#### 5.2.1. S-Propargyl-Cysteine (SPRC)

SPRC, a hydrogen sulfide donor, suppresses inflammatory signaling, including JAK2/STAT3; suppresses M1 macrophage polarization; and promotes M2 polarization, thereby establishing an anti-inflammatory microenvironment [10]. These effects are associated with reduced MMP-3/9/13 expression in condylar chondrocytes and attenuation of ECM degradation, supporting a cartilage-protective role in TMJOA lesions [10]. Although JAK–STAT inhibition is increasingly recognized as beneficial in OA, SPRC has been evaluated only in preclinical TMJOA models. Further TMJ-focused studies should define TMJ-specific efficacy, optimal dosing, and durability of macrophage repolarization in TMJ tissues.

#### 5.2.2. MSC-Derived Exosomes

MSC-derived exosomes and secretomes contain bioactive microRNAs and proteins that support chondrocyte survival and matrix homeostasis. In TMJOA models, intra-articular MSC-derived exosomes reduce inflammation, alleviate early pain and synovitis, increase chondrocyte proliferation and matrix synthesis, and improve cartilage and subchondral bone architecture [29]. These effects have been partly attributed to CD73-mediated adenosine generation and adenosine receptor-dependent activation of AKT, ERK, and AMP-activated protein kinase (AMPK) signaling [29]. Translation requires standardized production, optimized dosing intervals, and evaluation of long-term safety and TMJ-specific structural and functional endpoints.

#### 5.2.3. PTH1R-Mediated MSC Modulation

Parathyroid hormone (PTH) regulates osteogenesis via parathyroid hormone 1 receptor (PTH1R)-dependent cyclic adenosine monophosphate (cAMP)–protein kinase A (PKA) and phospholipase C (PLC)–protein kinase C (PKC) signaling, with signaling duration and subcellular localization influencing the balance between bone formation and resorption [118]. Preclinically, PTH(1–34) activates the PTH1R–cAMP–PKA–cAMP response element-binding protein (CREB) axis in orofacial bone marrow-derived MSCs, enhancing alkaline phosphatase (ALP), Runt-related transcription factor 2 (*RUNX2*), and collagen type I alpha 1 chain (*COL1A1*) expression and promoting mineralization. It also attenuates age-related temporomandibular joint osteoarthritis (TMJOA) changes, including reduced MMP-13 and collagen X expression in condylar cartilage [30]. Direct TMJ application of PTH1R-preactivated MSCs or their exosomes has rarely been investigated. Further TMJ-focused studies are needed to establish formulation strategies, dosing schedules, and tissue-specific safety profiles.

#### 5.2.4. Placenta-Derived Exosomes

Human placenta-derived exosomes (pExo) reduce pain and cartilage damage in preclinical knee OA models, with repeated dosing producing greater cartilage preservation than single administration [119]. In vitro, pExo promotes chondrocyte proliferation and migration, activates CREB and ERK1/2, and suppresses IL-1β-induced expression of MMP-8/13, inducible nitric oxide synthase (*NOS2*), and IL-6 [119]. TMJ-specific data are lacking; therefore, validation in TMJOA/TMID models, along with standardized manufacturing and safety testing, is required.

#### 5.2.5. GLP-1R Agonists

The glucagon-like peptide-1 receptor (GLP-1R) agonist exenatide activates the GLP-1R–G protein alpha s (Gs)–cAMP/PKA–p38 mitogen-activated protein kinase beta (p38β)–CREB axis in spinal microglia, inducing IL-10 and increasing M2-associated markers, while interleukin-10 receptor (IL-10R)–STAT3 signaling enhances β-endorphin expression and produces antinociceptive effects [120]. This GLP-1R–IL-10-mediated axis suggests a potential interface between immune modulation and pain-related signaling; however, its relevance to TMJ-local immuno-mechanical circuitry requires direct validation. In addition, the current evidence derives primarily from central nervous system models, and TMJ-specific preclinical validation is required.

#### 5.2.6. Artificial Cell-Derived Vesicles (ACDVs)

ACDVs are cell-free extracellular vesicle (EV) mimetics that preserve cartilage matrix, enhance chondrocyte proliferation and migration, restore chondrogenic markers, and improve subchondral bone microstructure in TMJOA models [121]. In vitro, ACDVs suppress IL-1β-induced apoptosis; restore *SOX9* and *COL2A1* expression; and downregulate TNF-α-associated, NF-κB-associated, and chemokine-related pathways [121]. Further TMJ-focused studies should define physicochemical properties, dosing strategies, and long-term safety in TMJ contexts.

#### 5.2.7. Strontium-Enhanced Exosomes

Strontium pretreatment enhances Alix-mediated selective miRNA loading and increases exosome yield in synovial mesenchymal stem cells (SMSCs) [122]. Sr-enhanced SMSC-derived exosomes exhibit superior therapeutic efficacy in TMJOA models, reducing cartilage matrix damage, promoting chondrocyte proliferation and migration, restoring chondrogenic markers, and accelerating recovery of pain thresholds, partly through mitigation of chondrocyte ferroptosis and reduction in osteoclast-associated pain [122]. Given that current evidence is limited to early preclinical studies, TMJ-specific biodistribution, dosing, and safety require further investigation.

#### 5.2.8. Fibrocartilage Stem Cell Niche Modulation

Located in the superficial zone of condylar fibrocartilage, fibrocartilage stem cells (FCSCs) play a critical role in cartilage development, maintenance, and repair. Experimental studies demonstrate that modulation of the local stem-cell niche—particularly through Wnt and Notch signaling—or direct FCSC transplantation can partially restore cartilage and subchondral bone defects in TMJ models [123]. In addition, age-related alterations in niche composition, including changes in growth factor availability, are associated with age-related degeneration and osteoarthritis of the condylar cartilage [124]. Future studies should further define FCSC-specific markers, lineage commitment, and the long-term stability and safety of niche-based regenerative strategies in TMJ tissues.

#### 5.2.9. Polydeoxyribonucleotide (PDRN)

PDRN induces anti-inflammatory and regenerative responses via adenosine A_2_A receptor signaling, reducing TNF-α and IL-1β and increasing IL-10 in multiple inflammatory models [125,126]. A_2_A–cAMP-linked modulation of PI3K/AKT and VEGF/VEGFR2 signaling may support tissue repair [125,126]. Clinically, PDRN injection has been associated with improvements in pain and mouth opening in patients with TMJ disorders, although evidence is retrospective and mechanistic data are lacking [127]. TMID-oriented validation should assess immune cell polarization, multi-tissue immuno-mechanical network effects, and structure–function outcomes.

### 5.3. Future Directions: Quantitative Validation and Clinical Translation

Future refinement of the TMID framework will benefit from the integration of systems biology and computational modeling approaches, including network-based analyses, quantitative molecular descriptors, and biomarker-based signatures enabling the transformation of complex molecular signaling patterns into measurable and analyzable data for disease stratification and predictive modeling, as well as descriptor-based computational approaches, including frameworks such as the Molecular Descriptor Family (MDF), which may further support the integration of structural and functional properties of signaling molecules into predictive models and facilitate the translation of immuno-mechanical signaling patterns into actionable therapeutic insights, as well as ligand–receptor interaction modeling, with potential incorporation of atomistic and geometry optimization approaches. Such approaches may enable the transition from a conceptual, pathophysiology-oriented framework to a quantitatively validated and predictive model, thereby facilitating precision diagnostics and therapeutic development.

TMID does not redefine the molecular components of TMJ disease but rather redefines how these components are integrated, temporally organized, and interpreted within an immuno-mechanical disease framework. In this regard, TMID represents a disease-activity-oriented conceptual advance that moves beyond descriptive aggregation toward a mechanism-based interpretation of TMJ pathology. Critically, this framework enables the identification of stage-dependent immune–mechanical states, as well as the prediction of transitions between these states under varying mechanical and inflammatory conditions, and provides a basis for distinguishing biologically active disease from structurally similar but immunologically quiescent conditions—capabilities that are not captured by conventional structure-based or pathway-isolated approaches. Importantly, this framework does not assume immune activation as a universally primary driver but, rather, conceptualizes it as a context-dependent and potentially self-sustaining component within a dynamic, bidirectional feed-forward system. Accordingly, TMID does not impose a fixed causal hierarchy, as current evidence—largely derived from cross-sectional and associative studies—remains insufficient to definitively establish temporal or causal primacy, and will require TMJ-specific longitudinal and interventional investigations for clarification. Instead, the framework generates testable predictions regarding the temporal coupling between mechanical stress and immune activation, including coordinated cytokine signatures (e.g., IL-1β, IL-6, and TNF-α), shifts in macrophage polarization (M1-like to M2-like states), and alterations in T cell balance (e.g., the Th17/Treg axis), together with stage-dependent transitions linking inflammation to extracellular matrix degradation and subchondral bone remodeling. In addition, the framework predicts divergence in clinical trajectories and treatment responses among patients with comparable structural TMJOA findings, reflecting underlying differences in immune–mechanical activity states. While TMID does not yet provide standardized diagnostic thresholds, it establishes a falsifiable framework in which the absence of these coordinated molecular patterns, failure to demonstrate stage-dependent transitions, or lack of differential therapeutic responses across predicted disease states would directly challenge its core mechanistic assumptions. In this way, TMID supports the development of quantifiable, biomarker-guided classification systems, longitudinal validation strategies, and mechanism-based therapeutic stratification. Although the present framework is conceptual in structure, it is grounded in experimentally measurable signaling components and pathway-level interactions, providing a basis for future quantitative and computational formalization; importantly, while developed at the tissue- and cellular-level, further refinement of immuno-mechanical signaling mechanisms may benefit from integration with atomistic and computational modeling approaches, particularly geometric optimization of ligand–receptor interactions and structure-based modeling, which may provide a quantitative basis for linking mechanical stimuli to molecular signaling behavior and support the development of predictive, mechanistically grounded extensions of the TMID framework.

Importantly, TMID does not aim to supplant existing diagnostic systems or provide immediate clinical algorithms but, rather, to reshape clinical reasoning by linking structural findings with underlying immuno-mechanical disease activity. In contrast to conventional DC/TMD frameworks, which primarily guide diagnosis and management based on structural findings and symptom-based classification, TMID introduces a mechanism-oriented interpretive layer that enables clinicians to distinguish biologically heterogeneous disease states within structurally similar TMJOA presentations. This distinction allows for a shift from uniform, structure-based management toward mechanism-informed clinical reasoning, in which patients with comparable imaging findings may be stratified into immune-active versus mechanically dominant phenotypes. Such stratification may guide differential therapeutic considerations, including the preferential use of anti-inflammatory interventions in immune-active states and load-modifying or biomechanical approaches in mechanically dominant conditions. In this context, TMID does not replace DC/TMD-based decision-making but refines and extends it by incorporating inflammatory activity, immune status, and remodeling dynamics into the clinical interpretation of TMJ disease. Importantly, although certain inflammatory mediators may overlap with those observed in antigen-specific autoimmune arthritides, these cytokine profiles are not disease-specific and, therefore, do not constitute primary diagnostic determinants; rather, reliable clinical distinction depends on initiating context, immune specificity, and the presence or absence of systemic features. Accordingly, in clinical application, this framework requires the exclusion of systemic features, serologic markers (e.g., rheumatoid factor (RF), anti-cyclic citrullinated peptide (anti-CCP) antibodies), and multi-joint involvement, thereby ensuring that TMID is applied only within the context of mechanically driven, localized TMJ pathology.

## 6. Conclusions

TMID is proposed as a complementary pathophysiology- and mechanism-oriented conceptual framework that neither replaces the DC/TMD Axes I–III nor extends to systemic antigen-specific autoimmune arthritides. Instead, it provides a mechanistic interpretive framework that complements existing classification systems by integrating immune–mechanical signaling in mechanically driven temporomandibular joint pathology.

DC/TMD Axis I provides structure- and symptom-centered categories based on pain reports, palpation findings, functional examination, and imaging features; however, it does not directly reflect underlying pathogenic mechanisms or dynamic disease activity, thereby limiting its utility for early-stage identification and mechanism-informed intervention design [128]. Axis II, originally developed within the Research Diagnostic Criteria for Temporomandibular Disorders (RDC/TMD) and retained in DC/TMD, remains essential for assessing psychosocial and behavioral factors; yet, similarly, it does not enable mechanism-based disease staging [129,130]. In the current DC/TMD system, Axis III—which aims to incorporate systemic health and biological markers—remains under development, and recent recommendations explicitly emphasize the need for etiologic- and mechanism-based diagnostic algorithms and categories [131]. Consistent with this need, multiple biomarkers—including IL-1β, IL-6, TNF-α, PGE_2_, MMPs, and oxidative stress markers—have been associated with TMD pathophysiology and pain persistence; however, the heterogeneity of the evidence and a lack of standardization currently preclude their routine diagnostic or prognostic application [132].

To address these limitations, TMID conceptualizes the TMD spectrum as a pathologic continuum encompassing early immune activation through progressive fibrotic–erosive stages within an integrated osteoimmune framework. Core elements include regulation of M1–M2 macrophage polarization during immune-active phases [10]; RANKL/osteoclast-mediated bone resorption amplified by Th1/Th17–Treg imbalance [15]; subchondral bone degeneration linked to RANKL/OPG imbalance and inflammatory cytokines; and cytokine-associated pain mechanisms [16]. In parallel, repetitive mechanical overload induces ECM damage, leading to DAMP release and activation of TLR/RAGE signaling. These events amplify NF-κB and MAPK pathways, as well as integrin–FAK–ERK and PI3K signaling, resulting in upregulation of MMPs and COX-2. Collectively, these processes establish an immuno-mechanical feed-forward loop that promotes matrix degradation and tissue injury [5]. Additional evidence indicates that aberrant mechanosensing and altered mechanotransduction within the condylar cartilage–subchondral bone unit contribute to degenerative remodeling [9], while activation of the ROCK–actin axis is associated with synovial fibrotic changes in the TMJ [11].

By integrating convergent immunologic and mechanical pathways, TMID links clinical phenotypes to their underlying pathophysiologic mechanisms, revealing dimensions of disease activity not captured by structure-centered diagnostic axes. Importantly, this framework retains DC/TMD Axes I and II while conceptually superimposing mechanism-based disease stages and activity states. In this way, TMID establishes a conceptual platform for refined disease stratification and the rational development of mechanism-based therapeutic strategies within established diagnostic frameworks.

## Figures and Tables

**Table 1:** Conceptual working stages of temporomandibular immunologic disease (TMID).

Stage (Conceptual)	Dominant Features
Immune-active	damage-associated molecular patterns (DAMPs), macrophage polarization, cytokine amplification, early T cell involvement
Fibrotic–erosive	extracellular matrix (ECM) degradation, fibrosis, osteoclast activation, subchondral remodeling
Resolution/regenerative (context-dependent)	Attenuated inflammation, partial tissue repair

Note: This table outlines a provisional disease continuum of TMID, integrating immunologic activation, tissue remodeling, and structural alterations reported in temporomandibular joint osteoarthritis (TMJOA) and related arthropathies. These stages are proposed as a working framework to facilitate mechanistic interpretation, rather than as a definitive clinical staging system or diagnostic classification.

**Table 2:** Comparative immunopathological and mechanistic features of RA, PsA, OA, TMJOA, and TMID.

Category	RA	PsA	OA	TMJOA	TMID
Initiating driver	Antigen-specific adaptive immunity	Interleukin-23–interleukin-17–tumor necrosis factor axis; enthesitis	Aging; mechanical load; DAMPs	Mechanical overload; immune-active features	Mechanical overload; innate + non-antigen-specific adaptive immunity
Antigen specificity	High	Partial/mixed	Low	Low (non-antigen-specific)	Low (non-antigen-specific)
Immuno-mechanical coupling	Low	Intermediate	Low (weakly coupled)	High	High
Major immune cells	CD4^+^ T cells; B cells	Th17, γδ T, CD8^+^ T cells	Macrophages (innate-dominant)	M1-like macrophages; Th1/Th17	M1-like macrophages; Th1/Th17
Main therapeutic targets	Cytokine inhibition	Cytokine inhibition	Load reduction; symptom control	Immunomodulation + load control	Stage-specific: immunomodulation + mechano-pathway targeting

Note: This comparison highlights initiating drivers, antigen specificity, immuno-mechanical coupling, dominant immune cell profiles, therapeutic targets, and the conceptual scope of existing disease models across inflammatory and degenerative joint disorders. Abbreviations: RA, rheumatoid arthritis; PsA, psoriatic arthritis; OA, osteoarthritis; TMJOA, temporomandibular joint osteoarthritis; TMID, temporomandibular immunologic disease; DAMPs, damage-associated molecular patterns; CD, cluster of differentiation; Th17, T helper 17 cells; γδ T cells, gamma delta T cells; M1-like macrophages, classically activated macrophage-like phenotype.

**Table 3:** Structured summary of key mechanical drivers, immune mediators, convergent signaling pathways, and downstream outputs across the TMID framework.

Category	Key Mechanical Drivers	Key Immune Mediators	Convergent Signaling Pathways	Major Downstream Outputs	Stage Relevance
Initiation (damage sensing)	Repetitive loading; disc displacement-associated loading; osteochondral microdamage; ECM fragmentation	DAMPs (ECM fragments, HMGB1); PRRs (TLR2/4, RAGE); synovial macrophages	NF-κB (TLR–MyD88); MAPK	Synovial inflammation; TNF-α, IL-1β, IL-6 upregulation	Immune-active (early)
Immune amplification	Sustained loading; altered load distribution; ECM stiffening	M1 macrophages; Th1/Th17; TNF-α, IL-1β, IL-6, IL-17; CCL20–CCR6; CXCL9–11–CXCR3	NF-κB; JAK–STAT (STAT1/3); MAPK	Persistent synovitis; immune cell recruitment	Immune-active (progressive)
Matrix degradation and catabolic remodeling	Excessive loading; integrin-mediated mechanotransduction; osteochondral interface stress	MMPs (MMP-1/3/13); ADAMTS-4/5; cytokines; ROS	MAPK–AP-1; PI3K–AKT; NF-κB; integrin–FAK	ECM degradation; reduced *COL2A1*/aggrecan	Transition phase
Subchondral bone remodeling	Microcracks; increased condylar loading; osteochondral stress	RANKL/OPG imbalance; osteoclasts; macrophage–osteoclast coupling; IL-17, TNF-α	RANKL–NF-κB; MAPK; Wnt5a–Ror2–NFAT; PI3K–AKT	Bone resorption; erosion; osteophytes; sclerosis; cysts	Fibrotic–erosive
Fibrosis and mechanoadaptive remodeling	ECM stiffening; altered biomechanics	TGF-β; M2 macrophages; fibroblast remodeling; myofibroblasts	YAP/TAZ–TEAD; RhoA–ROCK–MRTF; PI3K–AKT; Smad	Fibrosis; angiogenesis; pathological remodeling	Fibrotic–erosive (late)
Resolution and regenerative (context-dependent)	Attenuated injurious inputs	M2 macrophages; Treg; IL-10, TGF-β; IGF-1	STAT3; TGF-β–Smad; NF-κB suppression	Inflammation resolution; tissue repair; reduced bone resorption	Resolution

Abbreviations: ECM, extracellular matrix; DAMPs, damage-associated molecular patterns; HMGB1, high mobility group box 1; PRRs, pattern recognition receptors; TLR, Toll-like receptor; RAGE, receptor for advanced glycation end-products; NF-κB, nuclear factor kappa-B; MAPK, mitogen-activated protein kinase; MyD88, myeloid differentiation primary response 88; JAK–STAT, Janus kinase–signal transducer and activator of transcription; STAT, signal transducer and activator of transcription; MMPs, matrix metalloproteinases; ADAMTS, a disintegrin and metalloproteinase with thrombospondin motifs; ROS, reactive oxygen species; PI3K, phosphoinositide 3-kinase; AKT, protein kinase B; FAK, focal adhesion kinase; AP-1, activator protein-1; RANKL, receptor activator of nuclear factor κB ligand; OPG, osteoprotegerin; Wnt, Wingless-related integrated site; Ror2, receptor tyrosine kinase-like orphan receptor 2; NFAT, nuclear factor of activated T cells; TGF-β, transforming growth factor beta; YAP, Yes-associated protein; TAZ, transcriptional coactivator with PDZ-binding motif; TEAD, TEA domain transcription factor; RhoA, Ras homolog family member A; ROCK, Rho-associated coiled-coil containing protein kinase; MRTF, myocardin-related transcription factor; Smad, SMAD family transcription factor; IGF-1, insulin-like growth factor 1; IL, interleukin; TNF-α, tumor necrosis factor alpha; CCL, C–C motif chemokine ligand; CCR, C–C motif chemokine receptor; CXCL, C–X–C motif chemokine ligand; CXCR, C–X–C motif chemokine receptor; Treg, regulatory T cells; Th, T helper cells; COL2A1, collagen type II alpha 1 chain.

## Data Availability

No new data were created or analyzed in this study. Data sharing is not applicable to this article.

## Supplementary Materials

The following supporting information can be downloaded at https://www.mdpi.com/article/10.3390/ijms27083363/s1.
