# Supplementary material for: Immuno-Mechanical Signaling Network Integration in Temporomandibular Joint Pathology: A TMID Conceptual Framework
Source: Int J Mol Sci. 2026 Apr 9;27(8):3363. doi: 10.3390/ijms27083363 (PMC13116888; doi:10.3390/ijms27083363)
Supplement: Supplementary file 1 [file ijms-27-03363-s001.zip › ijms-4217670-Supplementary Materials.pdf]

Supplementary Materials

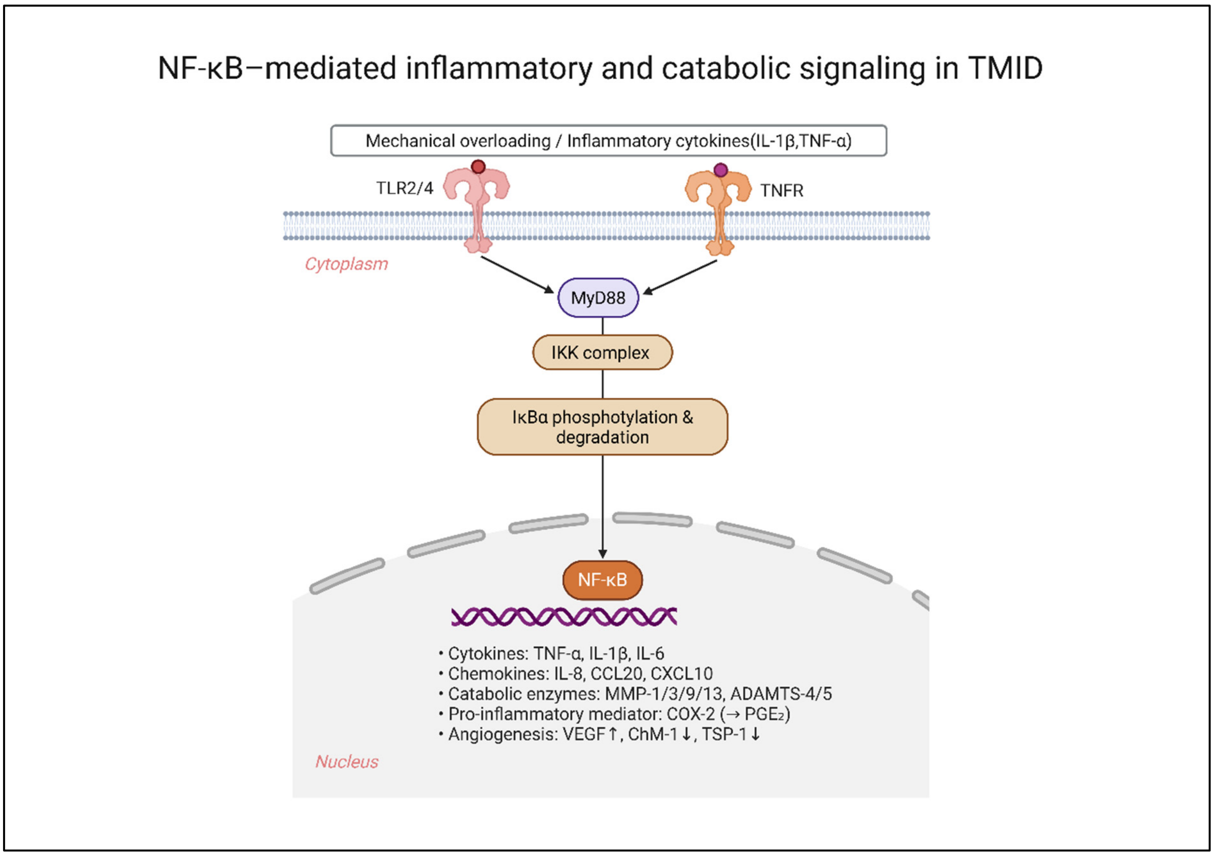

Supplementary Figure S1. NF-κB–mediated inflammatory and catabolic signaling in TMID. This schematic summarizes representative literature-based NF-κB signaling pathways in TMJ pathology. Mechanical overloading and inflammatory cytokines such as IL-1β and TNF-α activate TLR2/4 and tumor necrosis factor receptors (TNFR), converging on MyD88-dependent signaling and activation of the IKK complex. Subsequent phosphorylation and proteasomal degradation of IκBα promote NF-κB nuclear translocation and transcription of downstream inflammatory and catabolic genes. In TMJ tissues, NF-κB activation upregulates cytokines (TNF-α, IL-1β, IL-6), chemokines (IL-8, CCL20, CXCL10), matrix-degrading enzymes (MMP-1/3/9/13, ADAMTS-4/5), and COX-2–dependent PGE_2 production, and is associated with pathological angiogenesis characterized by increased VEGF expression and reduced levels of ChM-1 and TSP-1 [9,44,58,63,69,71,76]. Created in BioRender. KIM, H.

(2026) https://BioRender.com/i1k8xs9 (accessed on 1 March 2026).

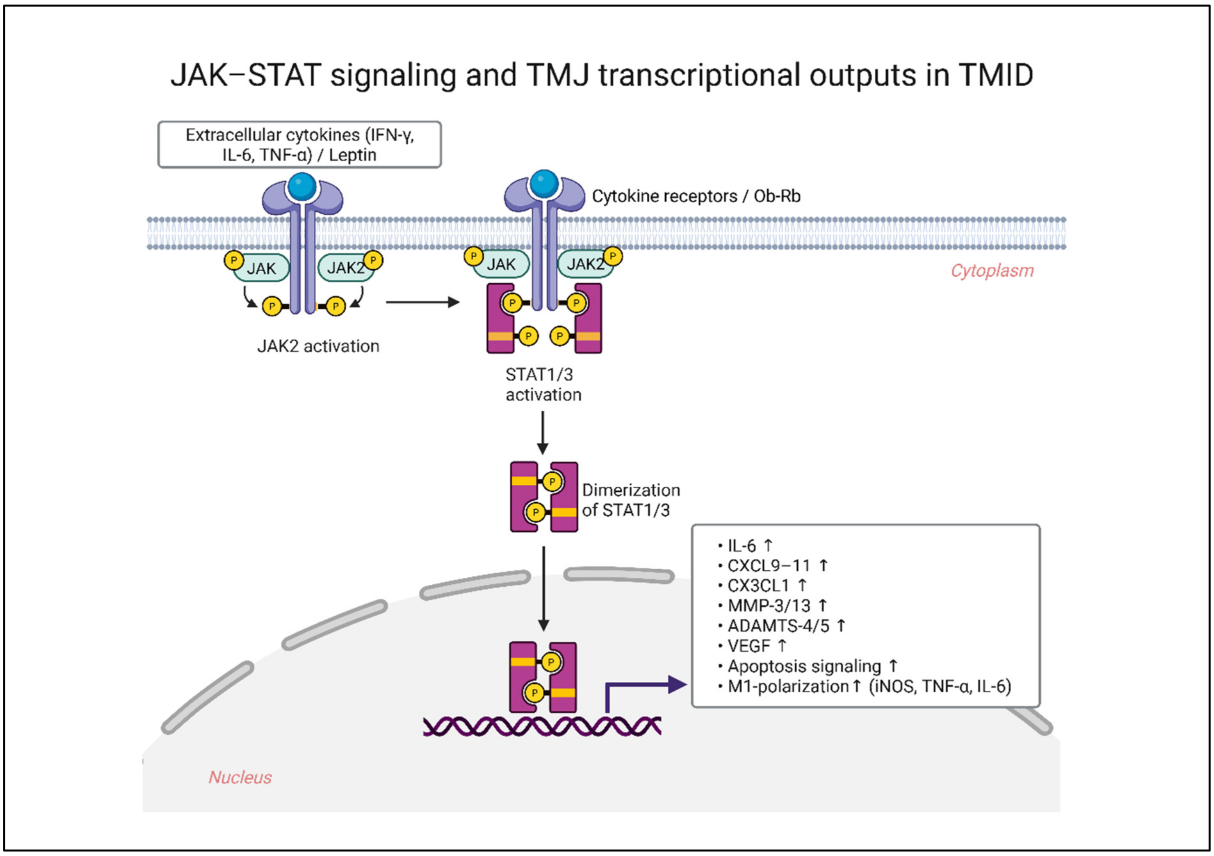

Supplementary Figure S2. JAK–STAT signaling and TMJ transcriptional outputs in TMID. This schematic summarizes representative literature-based JAK–STAT signaling pathways in TMJ pathology. Extracellular cytokines, including IFN-γ, IL-6, and TNF-α, as well as leptin, activate their respective receptors (cytokine receptors and Ob-Rb), leading to activation of JAK2 and subsequent phosphorylation of STAT1 and STAT3. Activated STAT1/3 undergo dimerization and nuclear translocation, resulting in transcriptional regulation of downstream inflammatory and catabolic genes. In TMJ tissues, JAK–STAT signaling promotes increased expression of cytokines (IL-6), chemokines (CXCL9–11, CX3CL1), matrix-degrading enzymes (MMP-3/13, ADAMTS-4/5), and angiogenic factors (VEGF), while also being associated with apoptosis signaling and M1 macrophage polarization characterized by upregulation of iNOS, TNF-α, and IL-6 [10,14,58,69,79,81]. Created in BioRender. KIM, H.

(2026) https://BioRender.com/i1k8xs9 (accessed on 1 March 2026).

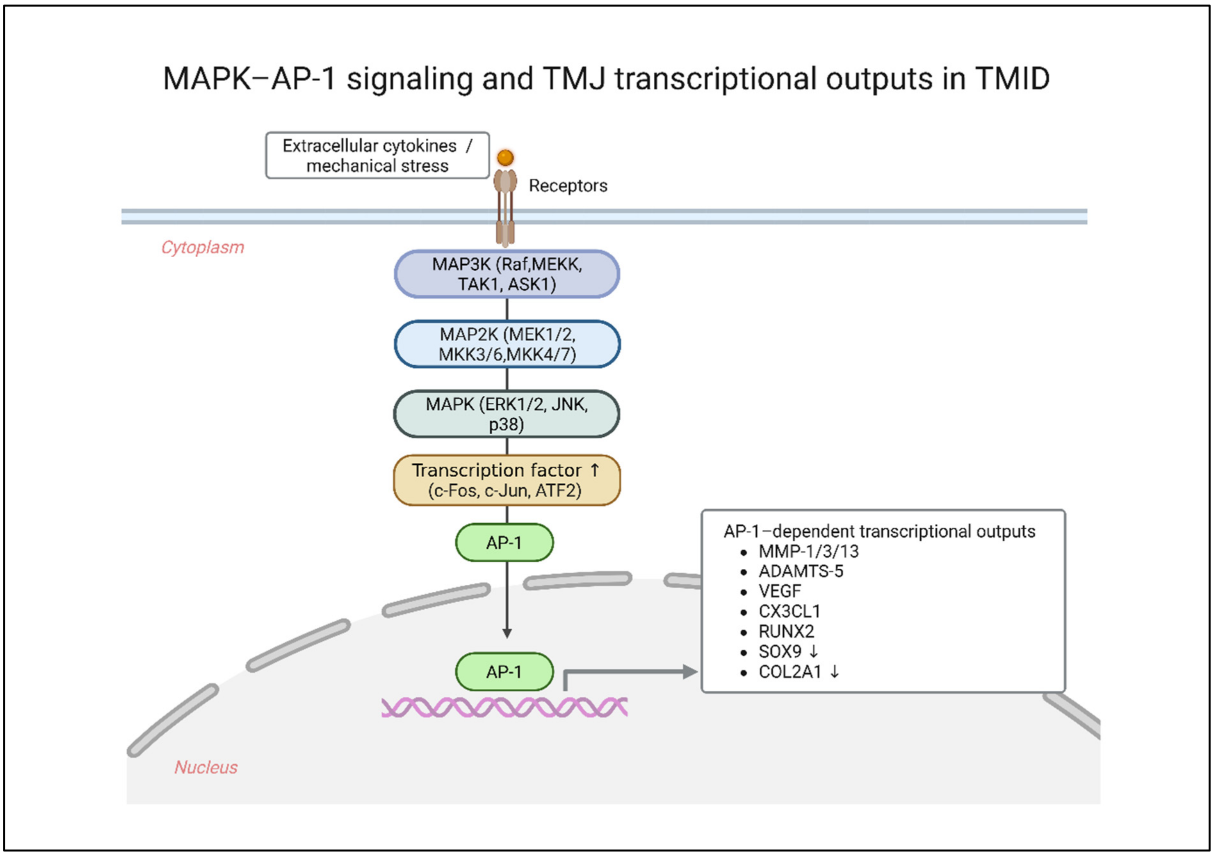

Supplementary Figure S3. MAPK–AP-1 signaling and TMJ transcriptional outputs in TMID. This schematic summarizes representative literature-based MAPK signaling mechanisms in TMJ pathology. Extracellular cytokines and mechanical stress activate MAPK cascades involving MAP3Ks (Raf, MEKK, TAK1, ASK1), MAP2Ks (MEK1/2, MKK3/6, MKK4/7), and MAPKs (ERK1/2, JNK, p38). Activated MAPKs phosphorylate c-Fos, c-Jun, and ATF2, leading to AP-1 activation. In TMJ tissues, AP-1 signaling induces upregulation of MMP-1/3/13, ADAMTS-5, VEGF, CX3CL1, and *RUNX2*, while suppressing *SOX9* and *COL2A1*, thereby contributing to extracellular matrix degradation, angiogenesis, and pathological tissue remodeling in TMID [5,64,69,71,81,83,84]. Created in BioRender. KIM, H. (2026) https://BioRender.com/i1k8xs9 (accessed on 1 March 2026).

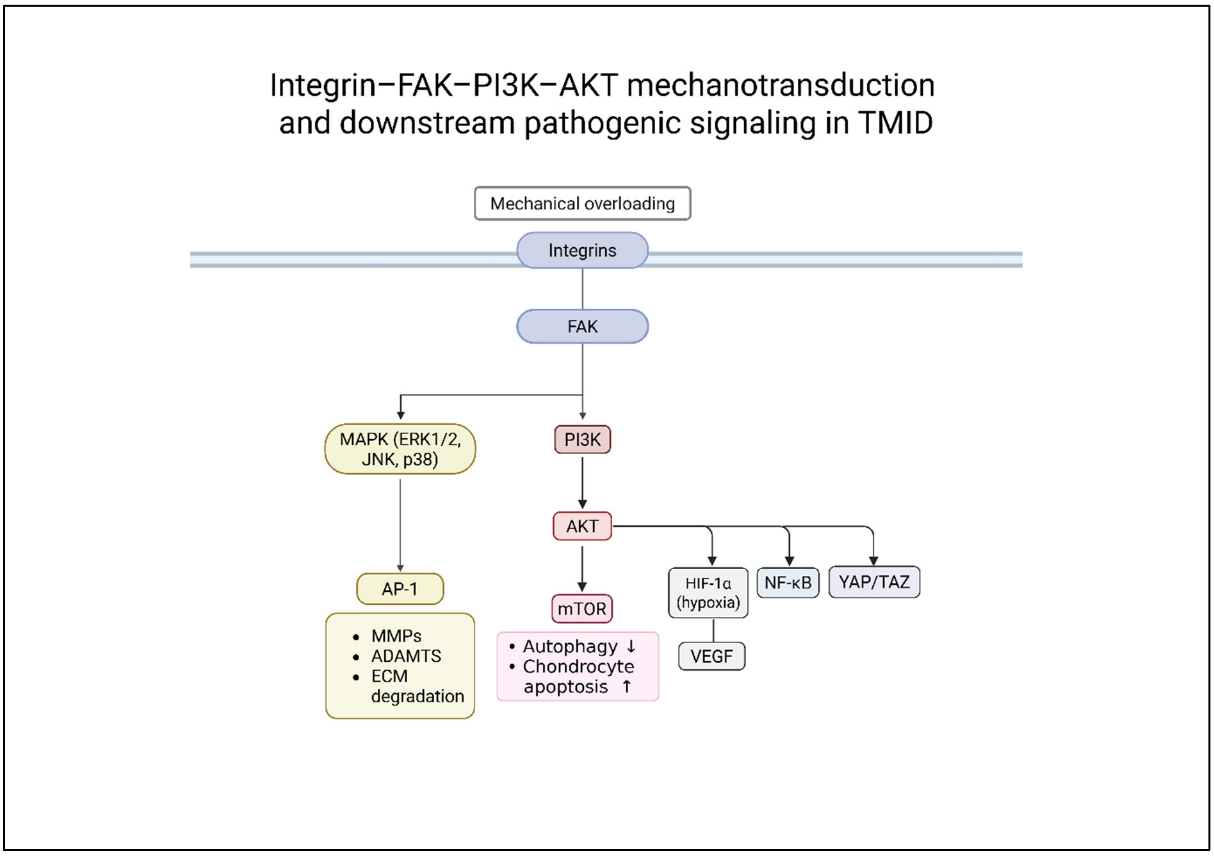

Supplementary Figure S4. Integrin–FAK–PI3K–AKT mechanotransduction and downstream pathogenic signaling in TMID. This schematic summarizes representative literature-based mechanotransduction signaling pathways in TMJ pathology. Mechanical overloading activates integrin-mediated FAK signaling, leading to parallel activation of MAPK (ERK1/2, JNK, p38) and PI3K–AKT pathways. MAPK signaling promotes AP-1–dependent transcription of matrix-degrading enzymes, including MMPs and ADAMTS, thereby contributing to ECM degradation. In parallel, PI3K–AKT signaling activates mTOR, suppressing autophagy and promoting chondrocyte apoptosis. Downstream signaling pathways functionally intersecting with the integrin–FAK–PI3K–AKT axis include NF-κB and YAP/TAZ, supported largely by mechanistic evidence derived from non-TMJ systems, as well as the mTOR–HIF-1α–VEGF axis, which has been directly implicated in hypoxia-associated angiogenic and degenerative responses in TMJ osteoarthritis [5,65,67,69,86,89]. Created in BioRender. KIM, H. (2026) https://BioRender.com/i1k8xs9 (accessed on 1 March 2026).

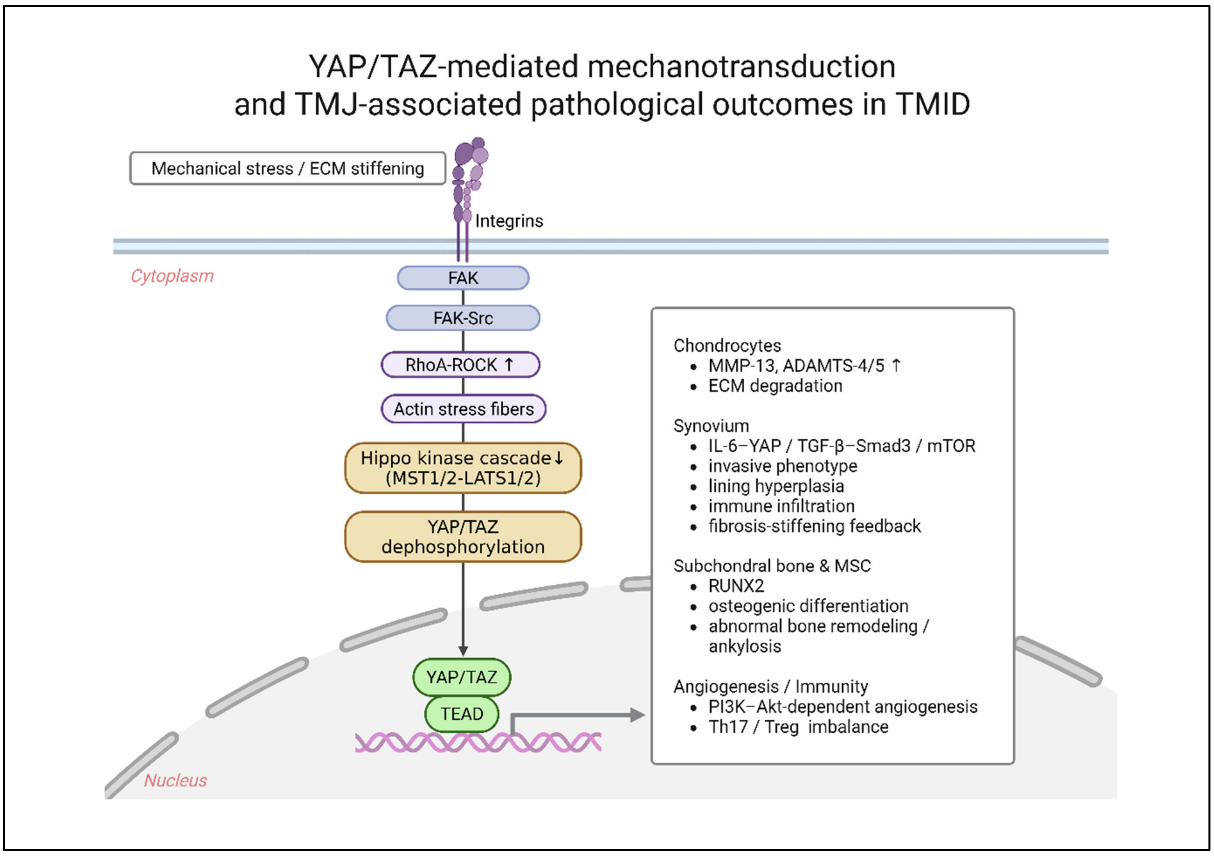

Supplementary Figure S5. YAP/TAZ-mediated mechanotransduction and TMJ-associated pathological outcomes in TMID. This schematic summarizes representative literature-based YAP/TAZ-dependent mechanotransduction pathways. The upstream integrin–FAK–RhoA–ROCK–Hippo signaling cascade depicts a generalized YAP/TAZ-dependent mechanotransduction pathway that has been primarily characterized in non-TMJ tissues. The downstream pathological outcomes summarized on the right represent TMJ-associated alterations reported in experimental and clinical studies, including ECM degradation in chondrocytes, synovial fibrotic remodeling, abnormal subchondral bone remodeling or ankylosis, and angiogenic and immune regulatory responses. Angiogenesis and immune imbalance shown in this schematic are supported in part by mechanistic evidence derived from non-TMJ systems and are presented here as functionally associated outcomes within TMJ pathological contexts [67,68,90,91,92]. Created in BioRender. KIM, H. (2026)

https://BioRender.com/i1k8xs9 (accessed on 1 March 2026).

Abbreviations

ADAMTS, a disintegrin and metalloproteinase with thrombospondin motifs;

AP-1, activator protein-1;

ASK1, apoptosis signal-regulating kinase 1;

ATF2, activating transcription factor 2;

CCL20, C-C motif chemokine ligand 20;

ChM-1, chondromodulin-1;

COX-2, cyclooxygenase-2;

CX3CL1, C-X3-C motif chemokine ligand 1;

CXCL, C-X-C motif chemokine ligand;

ECM, extracellular matrix;

ERK, extracellular signal-regulated kinase;

FAK, focal adhesion kinase;

HIF-1α, hypoxia-inducible factor 1 alpha;

IFN-γ, interferon gamma;

IKK, IκB kinase;

IL, interleukin;

iNOS, inducible nitric oxide synthase;

JAK, Janus kinase;

JNK, c-Jun N-terminal kinase;

MAPK, mitogen-activated protein kinase;

MAP2K, mitogen-activated protein kinase kinase;

MAP3K, mitogen-activated protein kinase kinase kinase;

MEK, mitogen-activated protein kinase kinase;

MEKK, mitogen-activated protein kinase kinase kinase;

MKK, mitogen-activated protein kinase kinase;

MMP, matrix metalloproteinase;

mTOR, mechanistic target of rapamycin;

MyD88, myeloid differentiation primary response 88;

NF-κB, nuclear factor kappa-B;

Ob-Rb, leptin receptor isoform b;

PGE_2, prostaglandin E_2;

PI3K, phosphoinositide 3-kinase;

ROCK, Rho-associated protein kinase;

RhoA, Ras homolog family member A;

RUNX2, runt-related transcription factor 2;

SOX9, SRY-box transcription factor 9;

STAT, signal transducer and activator of transcription proteins;

TAK1, transforming growth factor-β-activated kinase 1;

TLR, toll-like receptor;

TNF-α, tumor necrosis factor alpha;

TNFR, tumor necrosis factor receptor;

TMJ, temporomandibular joint;

TMID, temporomandibular immunologic disease;

VEGF, vascular endothelial growth factor;

YAP, Yes-associated protein;

TAZ, transcriptional coactivator with PDZ-binding motif.
